# *HIRA* loss transforms *FH*-deficient cells

**DOI:** 10.1126/sciadv.abq8297

**Published:** 2022-10-21

**Authors:** Lorea Valcarcel-Jimenez, Connor Rogerson, Cissy Yong, Christina Schmidt, Ming Yang, Monica Cremades-Rodelgo, Victoria Harle, Victoria Offord, Kim Wong, Ariane Mora, Alyson Speed, Veronica Caraffini, Maxine Gia Binh Tran, Eamonn R. Maher, Grant D. Stewart, Sakari Vanharanta, David J. Adams, Christian Frezza

**Affiliations:** ^1^ MRC Cancer Unit, Hutchison/MRC Research Centre, University of Cambridge, Cambridge CB2 0XZ, UK.; ^2^ CECAD Research Centre, University of Cologne, Joseph-Stelzmann-Str. 26, 50931 Cologne, Germany.; ^3^ Department of Surgery, University of Cambridge, Cambridge Biomedical Campus, Cambridge, UK.; ^4^ Cambridge University Hospitals NHS Foundation Trust, Cambridge, UK.; ^5^ Wellcome Sanger Institute, Wellcome Genome Campus, Hinxton, Cambridge, UK.; ^6^ School of Chemistry and Molecular Biosciences, University of Queensland, Molecular Biosciences Building 76, St. Lucia, QLD 4072, Australia.; ^7^ UCL Division of Surgery and Interventional Science, Specialist Centre for Kidney Cancer, Royal Free Hospital, Pond Street, London NW3 2QG, UK.; ^8^ Department of Medical Genetics, University of Cambridge, Cambridge, UK.; ^9^ Translational Cancer Medicine Program, Faculty of Medicine, Biomedicum Helsinki, University of Helsinki, Helsinki, Finland.; ^10^ Department of Physiology, Faculty of Medicine, University of Helsinki, Helsinki, Finland.

## Abstract

Fumarate hydratase (FH) is a mitochondrial enzyme that catalyzes the reversible hydration of fumarate to malate in the tricarboxylic acid (TCA) cycle. Germline mutations of *FH* lead to hereditary leiomyomatosis and renal cell carcinoma (HLRCC), a cancer syndrome characterized by a highly aggressive form of renal cancer. Although HLRCC tumors metastasize rapidly, FH-deficient mice develop premalignant cysts in the kidneys, rather than carcinomas. How *Fh1*-deficient cells overcome these tumor-suppressive events during transformation is unknown. Here, we perform a genome-wide CRISPR-Cas9 screen to identify genes that, when ablated, enhance the proliferation of *Fh1*-deficient cells. We found that the depletion of the histone cell cycle regulator (HIRA) enhances proliferation and invasion of *Fh1*-deficient cells in vitro and in vivo. Mechanistically, *Hira* loss activates MYC and its target genes, increasing nucleotide metabolism specifically in *Fh1*-deficient cells, independent of its histone chaperone activity. These results are instrumental for understanding mechanisms of tumorigenesis in HLRCC and the development of targeted treatments for patients.

## Teaser

*HIRA* loss transforms *FH*-deficient cells in vivo and in vitro, activating an H3.3-independent MYC transcriptional program.

## INTRODUCTION

Tumor initiation and progression require the metabolic rewiring of cancer cells (*1*, *2*). Fumarate hydratase (FH), a mitochondrial enzyme that catalyzes the reversible hydration of fumarate to malate in the tricarboxylic acid (TCA) cycle, has been identified as a bona fide tumor suppressor (*3*). FH loss predisposes to hereditary leiomyomatosis and renal cell carcinoma (HLRCC), a cancer syndrome characterized by the presence of benign tumors of the skin and uterus and a highly aggressive form of renal cancer (*4*). Its loss leads to aberrant accumulation of fumarate, an oncometabolite that drives malignant transformation (*5*, *6*). Although the link between FH loss, fumarate accumulation, and HLRCC is well known, the associated tumorigenic mechanism is still not fully understood (*7*). Although HLRCC tumors metastasize even when small, *Fh1*-deficient mice develop premalignant cysts in the kidneys, rather than overt carcinomas (*8*). These cysts are positive for the key tumor suppressor p21 (*9*). Because p21 expression is a central trigger of cellular senescence, it is postulated that this process could be an obstacle for tumorigenesis in *Fh1*-deficient cells. Consistent with this hypothesis, patients with HLRCC harbor the epigenetic suppression of p16, another key player of senescence (*10*). Here, we have confirmed that additional oncogenic events independent from a senescence bypass are required to allow full-blown transformation in *Fh1*-deficient cells. Moreover, a genome-wide CRISPR-Cas9 screen identified the histone cell cycle regulator (HIRA) as a target that, when ablated, increases proliferation and invasion in *Fh1*-deficient cells. HIRA, together with calcineurin binding protein 1 (CABIN1) and ubinuclein 1 (UBN1), is part of a histone chaperone complex that controls variant histone H3.3 deposition into the chromatin in a DNA replication–independent manner (*11*, *12*). Although its role in cancer is still poorly characterized, HIRA has been shown to suppress oncogene-induced neoplasia by activating a senescence phenotype in a mouse model of skin cancer and to block cell cycle progression in S phase in osteosarcoma cells (*13*, *14*). However, its role in HLRCC has not been previously described. Here, we found that *Hira*- and *Fh1*-deficient cells can form tumors with invasive features in the kidney in vivo. Notably, *Hira* depletion in *Fh1*-deficient cells controls the activation of an MYC- and E2F-dependent transcriptional and metabolic program, which is known to play different oncogenic roles during tumor initiation and progression (*15*, *16*). Notably, the activation of these programs is independent of H3.3 deposition into the chromatin, known to be controlled by HIRA (*12*). Overall, we have defined a previously unidentified oncogenic event occuring in FH-deficient tumors, which study will be instrumental for understanding the mechanisms of tumorigenesis in HLRCC and the development of targeted treatments.

## RESULTS

### *Fh1* loss impairs two-dimensional growth and enhances migration and invasion

**Fig. 1.**
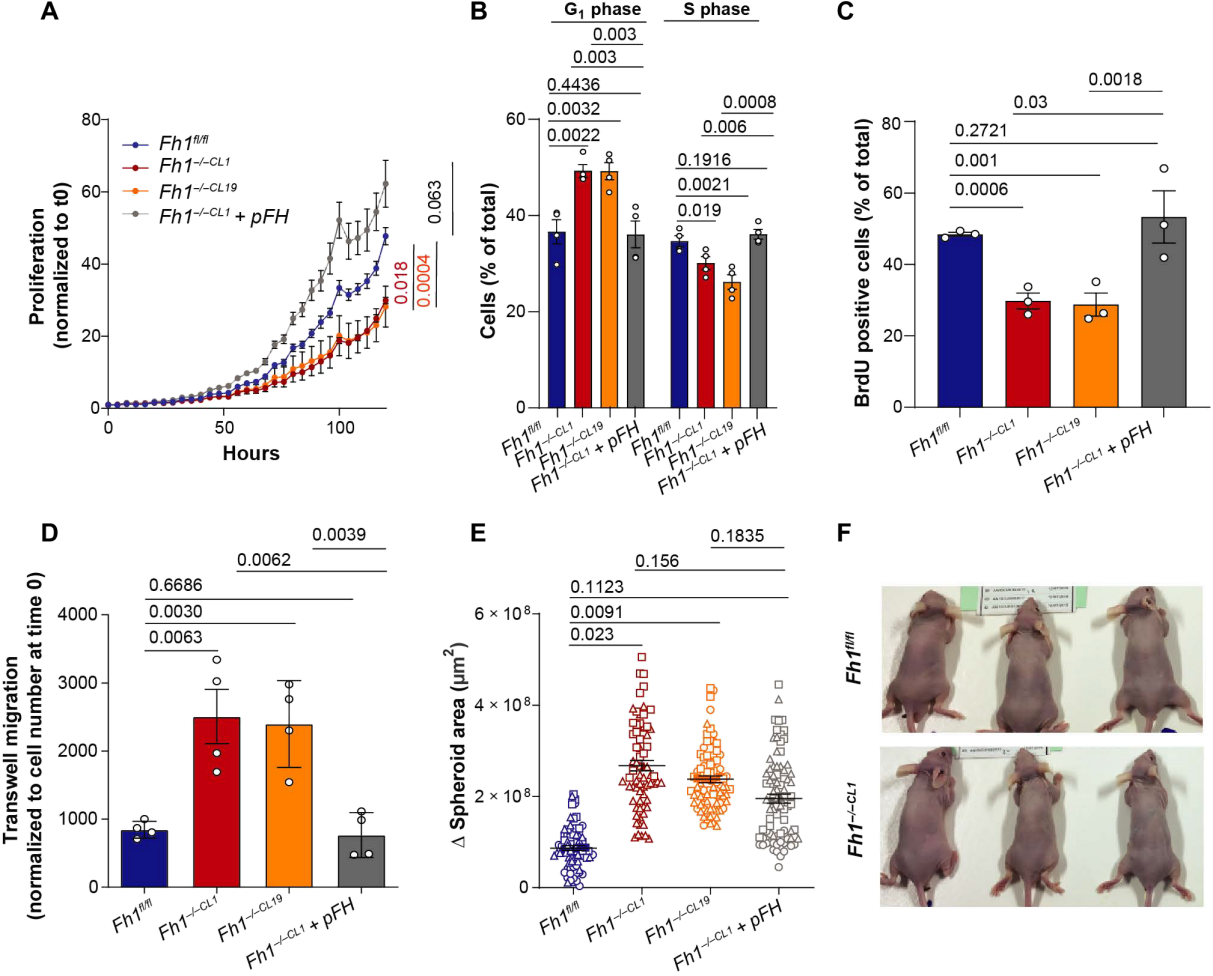
*Fh1* loss in kidney epithelial mouse cells compromises proliferation enhancing migration and invasion. (**A**) 2D growth measured using the Incucyte system of *Fh1*-proficient (*Fh1^fl/fl^*), *Fh1-*deficient (*Fh1^−/−CL1^* and *Fh1^−/−CL19^*), and *Fh1*-reconstituted (*Fh1^−/−CL1^ + pFH*) cell lines (*n* = 4). Data were normalized to time 0. The last time point was used for the statistical comparison. (**B**) Cell cycle comparisons were performed identifying differences between percentage of propidium iodide staining in G_1_ (physical cell growth interphase) and S (DNA synthesis) phases of cell cycle (*n* = 3 to 4). (**C**) DNA synthesis by means of BrdU incorporation into the DNA. Percentage of BrdU-positive cells relative to total nucleus number is plotted for each condition (*n* = 3). (**D**) Transwell migration of cells normalized by starting cell number (*n* = 4). (**E**) Analysis of increased spheroid area in 48 hours (*n* = 3). At least 20 spheroids were analyzed per experiment. Dots represent experiment 1, squares represent experiment 2, and triangles represent experiment 3. (**F**) Representative pictures of mice injected with *Fh1*-proficient (*Fh1^fl/fl^*) and *Fh1-*deficient (*Fh1^−/−CL1^*) cells (*n* = 5). No tumors were visible in any of the mice. Error bars represent SEM. Statistic tests performed: two-tailed Student’s *T* test (A, D, and E) and one-tailed Student’s *t* test (B and C). Numbers represent *P* value for all comparisons.

To investigate the oncogenic properties elicited by FH loss, we started by determining the two-dimensional (2D) growth, cell cycle profile, and migration properties of mouse *Fh1*-proficient (*Fh1^fl/fl^*), *Fh1*-deficient (*Fh1^−/−CL1^* and *Fh1^−/−CL19^*), and *Fh1*-reconstituted (*Fh1^−/−CL1^ + pFH*) epithelial kidney cell lines previously generated (*17*, *18*). *Fh1* loss led to a significant decrease in 2D growth, an arrest in G_1_ phase of the cell cycle, and a decrease in DNA synthesis determined by 5-bromo-2′-deoxyuridine (BrdU) incorporation into DNA (Fig.1, A to C, and fig. S1A). Notably, the overall decrease in cell proliferation was not associated with canonical senescence markers, such as β-galactosidase activity and *Cdkn1a/Cdkn2a* gene expression (fig. S1, B and C), contrary to previous observations in primary epithelial cells (*9*). Using a permeable derivative of fumarate, we confirmed that fumarate accumulation alone was sufficient to induce a senescence-independent decrease in cell proliferation (fig. S1, D to F). In contrast to the effect on proliferation, *Fh1* loss led to increased cell migration and invasive growth of spheroids embedded in collagen I matrix (Fig. 1, D and E, and fig. S1G), consistent with the epithelial-to-mesenchymal transition (EMT) activation that we recently showed (*18*). Notably, despite not being senescent and presenting invasive and migratory features, *Fh1*-deficient cells were unable to form xenografts in vivo (Fig. 1F). These results indicate that despite the higher invasion ability in vitro and the lack of senescence activation, *Fh1* loss in epithelial kidney cells is not sufficient to drive full-blown transformation. Therefore, we hypothesized that additional oncogenic events initiating transformation must occur in *Fh1*-deficient cells.

### *Hira* loss increases proliferation and invasiveness in *Fh1*-deficient cells

**Fig. 2.**
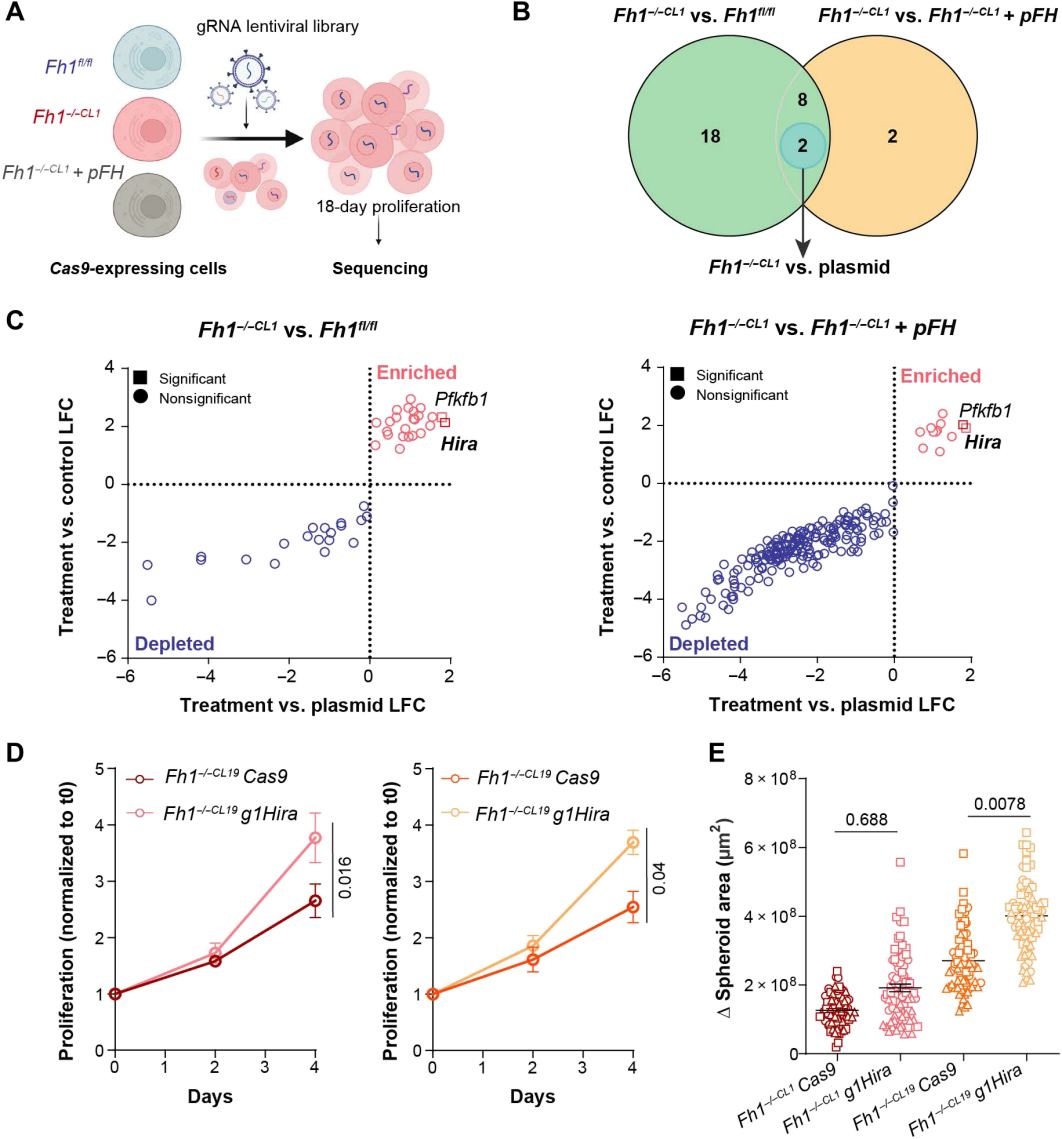
A genome-wide CRISPR-Cas9 screen identifies *Hira* loss as an oncogenic factor in *Fh1*-deficient cells. (**A**) Schematic of the CRISPR-Cas9 screen carried out. *Fh1*-proficient (*Fh1^fl/fl^*), *Fh1-*deficient (*Fh1^−/−CL1^*), and *Fh1*-reconstituted (*Fh1^−/−CL1^ + pFH*) cell lines expressing Cas9 were transduced with a pool mouse library containing ~90,000 guide RNAs (gRNAs) (*51*). Cells were grown for 18 days, and at least 90 million cells were harvested for consequent DNA extraction and high-throughput sequencing. (**B**) Venn diagram of the comparisons performed. Two enriched gRNAs dependent on *Fh1* and plasmid expression were identified (highlighted in blue). (**C**) Volcano plots showing the significantly depleted and enriched gRNAs for each comparison performed (*Fh1^−/−CL1^* versus *Fh1^fl/fl^*) and (*Fh1^−/−CL1^* versus *Fh1^−/−CL1^ + pFH*). Blue and pink colors represent the comparison between *Fh1* expression conditions. Shapes refer to whether a gene is significant in the comparison treatment condition versus plasmid. This comparison gets rid of significant depleted or enriched gRNAs depend on the corresponding gene basal expression in the cells. (**D**) 2D growth analysis of *Fh1*-deficient cells (*Fh1^−/−CL1^* and *Fh1^−/−CL19^*) under *Hira* depletion (*Fh1^−/−CL1^ g1Hira* and *Fh1^−/−CL19^g1Hira*) (*n* = 4). Data normalized to time 0. Statistics performed comparing the values of the last time point. (**E**) Representation of the increase in spheroid area for 48 hours (*n* = 3). At least 20 spheroids were analyzed per experiment. Dots represent experiment 1, squares represent experiment 2, and triangles represent experiment 3. Error bars represent SEM. Statistic tests performed: two-tailed Student’s *T* test (D) and one-tailed Student’s *t* test (E). Numbers represent *P* value for all comparisons. LFC, log_2_ fold change.

To identify genes that, when ablated, enhance proliferation in *Fh1*-deficient cells, we performed a genome-wide CRISPR screen (Fig. 2A) using *Fh1*-proficient (*Fh1^fl/fl^*), *Fh1*-deficient (*Fh1^−/−CL1^*), and *Fh1*-reconstituted (*Fh1^−/−CL1^ + pFH*) cells stably expressing Cas9. We transduced the cells with a genome-wide guide RNA (gRNA) mouse V2 CRISPR pooled library and propagated them for 18 days, as previously described (*19*, *20*). Analysis of the high-throughput sequencing data led to the identification of two significantly enriched gRNAs dependent on *Fh1* deficiency (Fig. 2B). One of them was the metabolic enzyme *Pfkfb1*, and the other one was *Hira* (Fig. 2C). Hira is part of a chaperone complex that controls the deposition of the H3.3 histone variant into chromatin. Notably, HIRA is known to regulate the epigenetic landscape of senescent cells, to suppress neoplasia (*13*, *14*), and, more generally, to play a role in tumorigenesis (*21*, *22*). Because of these early findings, we decided to investigate its role in *Fh1*-deficient cells.

We validated the screen by generating *Hira-*deficient cells and confirmed *Hira* depletion by RNA and protein expression, as well as the canonical markers of FH loss such as *Nqo1* and fumarate accumulation (fig. S2, A and B). Consistent with the screen results, *Hira* loss increased the 2D growth in two independent *Fh1*-deficient clones but had no effect in *Fh1*-proficient or *Fh1*-reconstituted cells when compared to control cell lines expressing the empty vector V2 (Cas9 cells) (Fig.2D and fig. S2C). *Hira* and *Fh1* loss were also associated with an increased percentage of cells in the S phase of the cell cycle and BrdU incorporation into the DNA (fig. S2, D and E). Next, we investigated whether *Hira* loss enhanced the migration and invasion ability of the cells. *Hira* loss further increased the transwell migration and wound healing specifically in *Fh1*-deficient, but not *Fh1*-proficient, cells (fig. S2, F and G). Furthermore, *Hira* loss led to an increased invasive growth of *Fh1*-deficient spheroids embedded in a collagen I matrix (Fig. 2E and fig. S2H). Notably, we confirmed these findings with an additional gRNA for *Hira* (g4Hira) (fig. S3, A to D). The specificity of the effects of *Hira* depletion was corroborated by rescuing Hira expression in the *Hira*- and *Fh1*-deficient cell lines, which led to a significant decrease in proliferation in all cell lines (fig. S3, E and F). Together, these results indicate that the loss of *Hira* in *Fh1*-deficient cells enhances proliferation, migration, and invasion and could play a significant role in the tumorigenesis associated with FH loss.

### *Hira*- and *Fh1-*deficient cells promote tumor initiation, growth, and invasion in vivo

**Fig. 3.**
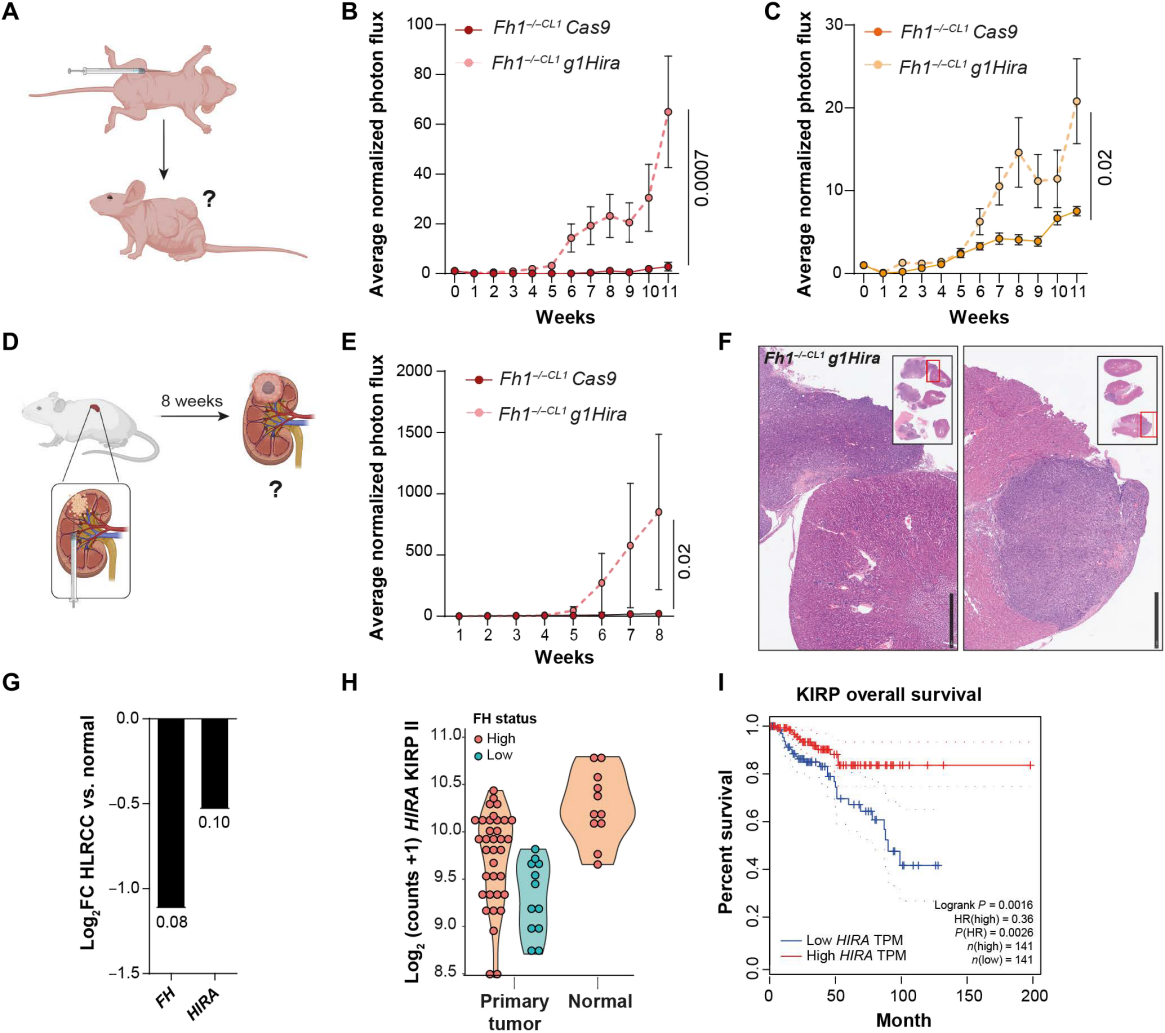
*Hira-* and *Fh1-*deficient cells promote tumor initiation, growth, and invasion in vivo. (**A**) Scheme of xenograft injections in the flank of nude mice. Two million cells were injected in each flank (5 mice, 10 injections in total), and tumor initiation/growth was monitored for 11 weeks by In Vivo Imaging System (IVIS) bioluminescence imaging (BLI). (**B** and **C**) Xenograft tumor growth by means of average BLI and flux intensity normalized to day 0 (*n* = 10 tumors) of *Hira*- and *Fh1*-deficient cells (*Fh1^−/−CL1^ g1Hira* and *Fh1^−/−CL19^ g1Hira*). (**D**) Scheme of orthotopic experiments carried out. Cells were injected in the kidney capsule (*n* = 4 kidneys per condition). Tumor initiation, growth, and invasion were analyzed for 8 weeks by IVIS BLI. (**E**) Representation of average luminescence signal by means of BLI and flux intensity normalized to day 1 (day after surgery) of *Fh1*-deficient (*Fh1^−/−CL1^ Cas9*) and *Hira-* and *Fh1*-deficient cells (*Fh1^−/−CL1^ g1Hira*). (**F**) Representative hematoxylin and eosin images of the kidney injected with *Hira*- and *Fh1*-deficient cells. Tumors attached to the kidney capsule and invasive lesions within the kidney can be observed. Small square represents the whole sections of the kidney and adjacent tumors. Scale bars, 1 mm. (**G**) Analysis of FH and HIRA expression data from patients with HLRCC (*25*). Numbers on the bars represent *P* values. (**H**) Gene expression of HIRA in KIRP II comparing normal and primary tumor samples. Tumor samples with low FH expression are represented in blue. (**I**) Overall survival data associated to HIRA expression from KIRP using Gene Expression Profiling Interactive Analysis (GEPIA) (*26*). Low/High HIRA represents top and bottom 50%. Error bars represent SEM. Statistic test performed: two-tailed Mann-Whitney *U* test. Numbers represent *P* value for all comparisons. TPM, transcripts per million, HR, hazard ratio; FC, fold change.

Given the role played by *Hira* depletion in *Fh1*-deficient cells in vitro, we next investigated its role in vivo. To do so, we performed two different in vivo experimental approaches. First, we xenografted 2 million cells subcutaneously per condition in the flanks of five nude mice and monitored tumor formation and growth for 11 weeks using a bioluminescence-based approach (Fig. 3A). *Hira*- and *Fh1*-deficient cells significantly increased tumor initiation and formation compared to *Fh1-*deficient cells only (Fig.3, B and C, and fig. S4A). Next, we performed orthotopic cell injections in the kidney capsule and monitored the survival and growth of the cells (*23*) (Fig. 3D). This approach better mimics the microenvironment where FH-deficient tumors develop in vivo (*24*). Notably, tumors were detected in the kidney capsules injected with *Hira*- and *Fh1*-deficient cells, while no tumors were observed in those injected with *Fh1*-deficient cells (Fig.3, E and F, and fig. S4, B and C). Cells deficient for *Hira* and *Fh1* showed invasive potential within the kidney (Fig. 3F), indicating a higher degree of malignancy. Notably, control cells (*Fh1^fl/fl^*) did not generate tumors in vivo (fig. S4, D and E) in either of the experimental approaches.

To corroborate these results in human samples, we took advantage of a previously published transcriptomics analysis of 25 patients with HLRCC (*25*). We confirmed the down-regulation of *FH* and *HIRA* expression in patients with HLRCC compared to matched normal tissue (Fig. 3G). Furthermore, we validated the down-regulation of *HIRA* gene expression in kidney biopsies from two additional patients with HLRCC compared to normal adjacent tissue (fig. S4F).

As HLRCC predisposes to Kidney renal papillary cell carcinoma type 2 (KIRP II), we analyzed *HIRA* expression in those tumors (*4*). In addition, we used the GEPIA tool to study the association of *HIRA* expression and overall survival in kidney renal papillary cell carcinoma (KIRP) (*26*). Notably, *HIRA* was down-regulated in primary tumors of KIRP II patients with low FH expression compared to normal tissue, and the down-regulation of *HIRA* was associated with poorer overall survival in patients with KIRP (Fig. 3, H and I). Together, these results confirm the previous in vitro results and highlight the loss of *HIRA* expression as an oncogenic event for FH deficiency in vivo.

### *Hira* loss in *Fh1*-deficient cells leads to an H3.3 deposition-independent MYC and E2F transcriptional program activation

**Fig. 4.**
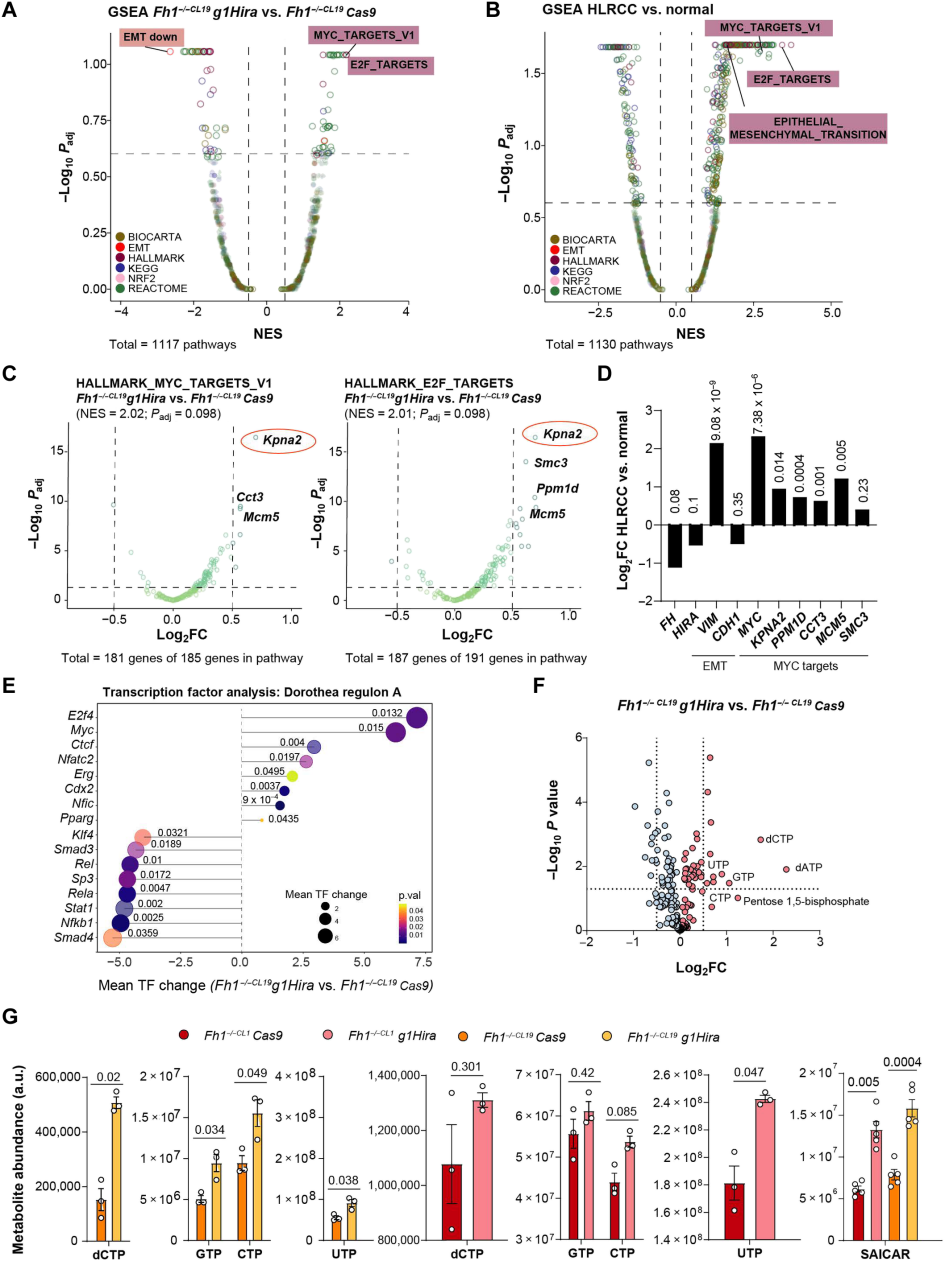
*Hira*- and *Fh1-*deficient cells activate an EMT program and Myc and E2f-target signatures. (**A**) Volcano plot representing the GSEA for *Fh1^−/−^g1Hira* versus *Fh1^−/−^* Cas9 cells. Signatures are colored depending on database represented. (**B**) Volcano plot representing the GSEA from the comparison between HLRCC and normal patient transcriptomic data. Signatures are colored depending on database represented. (**C**) Volcano plots of the genes present in the significantly up-regulated signatures in the GSEA for *Fh1^−/−CL19^ g1Hira* versus *Fh1^−/−CL19^ Cas9* cells (MYC_Targets_V1 and E2F_Targets). Orange circles on the top up-regulated target-*Kpna2*. (**D**) Transcriptomic expression of *FH*, *HIRA*, and EMT genes (*VIM* and *CDH1*) and MYC targets (*MYC*, *KPNA2*, *PPM1D*, *CCT3*, *MCM5*, and *SMC3*) in HLRCC patient cohort. Numbers over the bars represent *P* value. (**E**) Lollipop graph representing the mean TF change in the transcriptomic data comparing *Hira*- and *Fh1*-deficient cells with *Fh1*-deficient cells alone. (**F**) Volcano plot with the untargeted metabolomics performed. Nucleotides up-regulated in *Hira*- and *Fh1*-deficient cells. CTP, cytidine 5′-triphosphate; GTP, guanosine 5′-triphosphate; dATP, 2′-deoxyadenosine 5′-triphosphate; dCTP, 2′-deoxycytidine 5′-triphosphate; UTP, uridine 5′-triphosphate. (**G**) Metabolite abundance of the nucleotides shown for *Fh1*-deficient (*Fh1^−/-CL1/CL19^*) and *Hira*- and *Fh1*-deficient cell lines (*Fh1^−/−CL1/CL19^ g1Hira*) (*n* = 5). NES, normalized enrichment score. Error bars represent SEM. Cutoff for transcriptomic volcano plots: NES = ±0.5 and *P*_adj_ = 0.25 (=25%). Statistic test performed: two-tailed Student’s *T* test (G). For comparisons between *Fh1*-deficient cells and *Hira*- and *Fh1-*deficient cells, a paired comparison was performed. Numbers represent *P* value for all comparisons. a.u., arbitrary units.

To gain insight into the mechanism by which *Hira* loss promotes transformation in *Fh1*-deficient cells, we performed a transcriptomic analysis followed by a gene set enrichment analysis (GSEA) of *Hira*- and *Fh1*-deficient cells. This analysis showed an up-regulation of EMT, MYC, and E2F target signatures (Fig. 4A). These transcriptional changes were genotype specific because *Fh1* or *Hira* loss in *Fh1*-proficient cells did not elicit the up-regulation of these signatures (fig. S5, A to C). The activation of an EMT program in *Fh1*-deficient cells has been previously described by our group (*18*). *Hira* loss further enhanced the down-regulation of the epithelial marker E-cadherin and the up-regulation of the mesenchymal marker Vimentin by transcript and protein levels in *Hira*- and *Fh1*-deficient cells (*g1Hira* and *g4Hira*) and in the xenograft tumors (fig. S5, D to G). When we performed the GSEA in the HLRCC patient cohort, we observed MYC and E2F target signatures as the top up-regulated ones, together with a significant up-regulation of the EMT signature (Fig. 4B).

We then investigated the most up-regulated genes in MYC and E2F target signatures in *Hira*- and *Fh1*-deficient cells (Fig. 4C). The top up-regulated factor in both signatures was the karyopherin subunit alpha 2 (*Kpna2*), a nuclear transporter involved in the nucleocytoplasmic transport pathway of several tumor-associated proteins, including MYC and E2Fs (*27*). Notably, the overexpression of this transporter has been shown to promote cell proliferation (promoting the G_1_-S cell cycle transition), migration, invasion, and cell matrix adhesion in several cancers, including renal cell carcinoma (*27*, *28*). A DNA replication initiation factor [Minichromosome Maintenance Complex Component 5 (MCM5)] and protein phosphatase 1D (PPM1D), a gene encoding the PP2Cδ and negative regulator of cellular stress response pathways, were also up-regulated as part of these signatures (Fig. 4C) (*27*, *29*). We confirmed that the down-regulation of *FH* and *HIRA* was associated with the up-regulation of MYC and E2F targets, as well as the EMT activation, in the cohort of patients with HLRCC from the study by Crooks *et al.* (*25*) (Fig. 4D and fig. S6A). These transcriptional changes were not observed in *Hira*-deficient *Fh1*-proficient cells (fig. S6B) and were further validated with an additional gRNA for Hira (*g4Hira*) (fig. S6C) and in the *Hira*- and *Fh1*-deficient xenograft tumors generated in vivo (fig. S6D). Notably, the expression of these signatures was associated with poorer overall survival in patients with KIRP (fig. S6E). Moreover, a transcription factor (TF) analysis performed revealed E2F4 and MYC as two of the top TFs involved in the transcriptional activation led by *Hira* loss in *Fh1*-deficient cells, but this signature was not present in *Fh1*-deficient cells, when compared to their *Fh1*-proficient counterpart (Fig. 4E and fig. S6F). This validates the relevance of cell cycle progression and oncogenic transcriptional activation in the cells. Last, we confirmed the activation of MYC-dependent pathways through an untargeted metabolomics analysis. MYC activation has been previously associated with nucleotide biosynthesis (*30*). The metabolomics analysis showed a significant up-regulation of nucleotide-associated metabolites under *Hira* loss in two *Fh1*-deficient cell lines (Fig. 4, F and G), which validates the increase in proliferation previously shown (Fig. 2D).

**Fig. 5.**
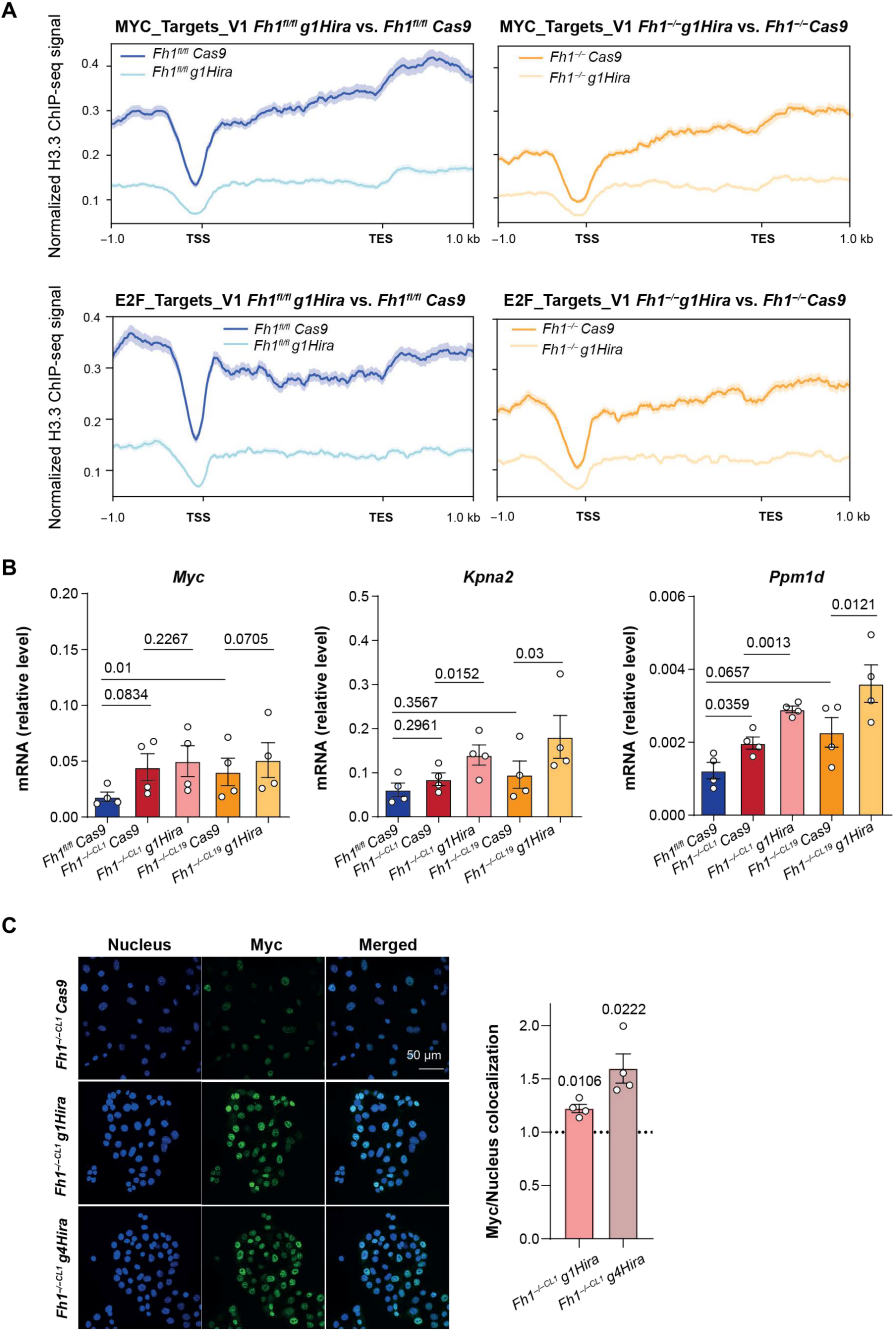
The activation of Myc and E2f target signatures is independent of H3.3 deposition. (**A**) Normalized ChIP-seq signal associated with MYC and E2F target signature expression for all conditions. Shadows represent the SEM. (**B**) Quantitative reverse transcription polymerase chain reaction showing expression levels for *Myc*, *Kpna2*, and *Ppm1d* in control (*Fh1^fl/fl^*), *Fh1*-deficient (*Fh1^−/−CL1^*), and *Hira*- and *Fh1*-deficient cells (*Fh1^−/−CL1/CL19^ g1Hira*) (*n* = 4). (**C**) Confocal representative images for Myc and nuclear (4′,6-diamidino-2-phenylindole) colocalization comparing the effect of two independent gRNAs for *Hira* (*g1Hira and g4Hira*) in *Fh1*-deficient cells (*Fh1^−/−CL1^*) (*n* = 3). Dotted line represents *Fh1*-deficient cells (*Fh1^−/−CL1^ Cas9*) as a control. β-Actin was used as a housekeeping gene. TSS, transcription starting site; TESs, transcription end sites. Error bars represent SEM. Statistic test performed: two-tailed Student’s *T* test. For comparisons between *Fh1*-deficient cells and *Hira*- and *Fh1-*deficient cells, a paired comparison was performed. Numbers represent *P* value for all comparisons.

We next studied how HIRA regulates the activation of MYC and E2F transcriptional signatures. As mentioned before, HIRA, together with UBN1 and CABIN1, is part of a chaperone complex that controls the deposition of the histone variant H3.3 into the chromatin (*11*). Therefore, we first assessed whether the activation of MYC-, E2F-, and EMT-associated transcriptional programs was due to a remodeling of H3.3 deposition in the chromatin in *Hira*- and *Fh1*-deficient cells. To this aim, we performed a chromatin immunoprecipitation sequencing (ChIP-seq) for H3.3 in *Fh1*-proficient and *Fh1*-deficient cells and analyzed the effect of *Hira* loss. We observed an overall decrease in H3.3 deposition in *Hira*-deficient cells, independent of the set of genes transcriptionally up-regulated or down-regulated in the RNA sequencing (RNA-seq) analysis from both *Fh1-*proficient and *Fh1-*deficient cells (fig. S7A). Then, we associated the expression of MYC, E2F, and EMT signatures with H3.3-normalized ChIP signal in *Fh1*-proficient and *Fh1*-deficient cells in the presence or absence of *Hira*. *Hira* loss resulted in a decreased H3.3 deposition into the chromatin associated to the expression of the signatures, independent of *Fh1* expression (Fig. 5A and fig. S7B). Moreover, no major differential changes were observed for H3.3 deposition in the promoter regions of the most up-regulated genes from the MYC transcriptional signature (fig. S7C). While the activation of MYC and E2F1 target signatures is specific of *Hira* loss in *Fh1*-deficient cells (Fig. 4A and fig. S5, A and B), the normalized H3.3 ChIP signal is decreased when *Hira* is loss in both *Fh1-*proficient and *Fh1-*deficient cells. Then, we conclude that HIRA loss may induce a transcriptional activation independent of its role controlling H3.3 deposition.

Notably, although gene expression analysis showed an up-regulation of Myc in both *Fh1*-deficient and *Hira*- and *Fh1*-deficient cells, this was not the case for its targets *Kpna2* and *Ppm1d*, whose up-regulation was specific of *Hira* loss (Fig. 5B), suggesting that Myc activity, rather than its expression, is increased by the loss of *Hira*. Consistent with this hypothesis, we observed that MYC localization in the nucleus was higher in *Hira*- and *Fh1*-deficient cells when compared to *Fh1*-deficient cells only (Fig. 5C). These results demonstrate that MYC transcriptional activity in *Fh1*-deficient cells may be hindered by Hira expression, compromising the activation of its transcriptional program, and that *Hira* loss promotes MYC binding to its transcriptional targets.

## DISCUSSION

Although germline mutations of FH loss predispose to renal cancer in patients with HLRCC, it is still unclear whether additional oncogenic events are required to transform *Fh1*-deficient cells. In this work, using a genome-wide CRISPR screening, we identified *Hira* as an oncogenic factor for *Fh1*-deficient cells in vitro and in vivo. We show that the loss of *Hira* is a prerequisite to the full activation of the proto-oncogene Myc, affecting its localization in the nucleus and its transcriptional activity (fig. S8). Although MYC-altered expression in FH-deficient cells has been hypothesized (*31*), the role of this oncogene in their transformation has not been fully investigated. The regulation of the Myc target *Kpna2* by *Hira* needs further exploration, and it would be interesting to investigate whether it occurs in other cancers, beyond HLRCC (*26*, *27*). Furthermore, while HIRA has been shown to interact with c-MYC on chromatin, our work reveals an essential regulatory function of Myc activity by *Hira*, which is independent of its role as an H3.3 chaperone (*32*). This confirms a noncanonical function of HIRA, independent of its chaperone role, as previously observed in another study (*33*). Further investigation is needed to fully assess whether this function occurs at the chromatin level or outside of the nucleus and whether it is dependent on the deposition of the noncanonical histone H3.3 by HIRA. HIRA is known to play a role in senescence, an established feature of primary FH-deficient cells triggered by fumarate. Moreover, HIRA can block the S-phase progression in the cell cycle, inhibiting DNA replication (*13*, *14*). It is therefore possible that upon FH loss, the replicative arrest that we observed is mediated by HIRA, at least in part by its suppressive role against Myc. Although we have not detected changes in Hira levels between *Fh1*-proficient and *Fh1*-deficient cells, the loss of FH may affect its function on chromatin. It will be important to determine whether FH loss and the accumulation of fumarate affect the binding of HIRA on chromatin or its H3.3 deposition activity. Overall, these results expand our understanding of how tumorigenesis occurs in *Fh1*-deficient cells and highlight the role of HIRA activating MYC-associated transcriptional programs that could lead to the development of targeted treatments.

### Limitations of the study

Although comprehensive in the study of *Hira* loss triggering transformation in *Fh1*-deficient cells, this study was performed in immortalized mouse epithelial cells. Further validations would be required in vivo, to ascertain the effect of *Hira* loss in an *Fh1*-deficient kidney-specific mouse model. Furthermore, it will be crucial to further investigate the mechanism by which HIRA modulates MYC- and E2F1-dependent transcriptional programs. In this study, we hypothesize that HIRA controls the access of MYC to its transcriptional targets at the chromatin level. Whether this control is directly or indirectly regulated by HIRA would be detrimental to understand its role in different tumors.

## MATERIALS AND METHODS

### Cell culture, cell line generation, and treatments

*Fh1*-proficient (*Fh1^fl/fl^*), *Fh1*-deficient (*Fh1^−/−CL1^* and *Fh1^−/−CL19^*), and *Fh1*-reconstituted (*Fh1^−/−CL1^ + pFH*) mouse cell lines were obtained as previously described (*17*, *18*). Senescence-positive cells were provided by M. Paez Ribes. Cells were cultured using high-glucose (4.5 g/liter) Dulbecco’s modified Eagle’s medium (DMEM) (Gibco-41966-029) supplemented with 10% heat-inactivated fetal bovine serum (FBS). Monomethyl fumarate (Sigma-Aldrich) powder was resuspended in dimethyl sulfoxide (Thermo Fisher Scientific) at 500 mM and used at 400 μM for 48 hours. Cells were transduced using a lentiviral packaging system with a Cas9-expressing vector (pKLV2-EF1a-Cas9Bsd, Addgene no. 68343), and Cas9 activity was measured using two different Cas9 reporters [pKLV2-U6gRNA5(gGFP)-PGKBFP2AGFP (Addgene no. 67980) and pKLV2-U6gRNA5(empty)-PGKBFP2AGFP (Addgene no. 67979)] using an LSRFortessa (BD Biosciences) fluorescence-activated cell sorter (FACS). The screen was performed using the mouse improved genome-wide knockout CRISPR library V2 [pKLV2-U6gRNA5(Bbs I)–PGKpuro2ABFP-W, Addgene no. 67988] and the different gRNAs used for *Hira* depletion were cloned into a vector with the same backbone as the library [pKLV2-U6gRNA5(Bbs I)–PGKpuro2ABFP-W, Addgene no. 67974]. The cells transduced with the backbone vector (indicated as *Cas9*) were used as a control for *Hira* depletion. All gRNA constructs used in this study were purchased from Sigma-Aldrich. The sequences used were *g1Hira* (ACATGTTTGAAACGGCCTC) and *g4Hira* (TAGGGAGCGGTTTCCCGCCG). The second one was designed using CRISPOR targeting exon 1 of *Hira* (http://crispor.tefor.net/). For the different *Hira* gRNAs, the total cell pool was used for the experiments, and no single clones were selected. Luciferase-expressing cells for in vivo experiments were generated using a Cherry-Luc construct provided by S.V.’s laboratory. Hira reexpression was induced using a mouse-tagged open reading frame clone from Origene (MR217357), and cells were transiently transduced using Lipofectamine 2000 as a transfection reagent (Thermo Fisher Scientific). Experiments were carried out 24 hours after transduction.

#### Lentiviral production and transduction

Human embryonic kidney (HEK) 293FT cells were transfected with the plasmid mix (plasmid of interest, PPAX2 and pMD2.G) using Lipofectamine 3000 (Thermo Fisher Scientific) as a transfection reagent diluted in Opti-MEM (Thermo Fisher Scientific). The medium containing the virus was collected 48 to 72 hours after transfection and filtered using a 0.45-μm sterile filter. Cells were transduced twice with the lentiviral supernatant in the presence of polybrene (8 μg/ml; Millipore). Selection of cells was performed 24 hours after transduction using puromycin (2 μg/ml) and blasticidin (10 μg/ml) both from Gibco/Thermo Fisher Scientific.

### Animals

All animal experiments were performed in accordance with protocols approved by the Home Office (UK) and the University of Cambridge ethics committee (PPL PFCB122AA). For xenograft subcutaneous injection, five 7-week-old female nonobese diabetic/severe combined immunodeficient mice obtained from Charles River Laboratories were injected per condition in each flank with 2 million cells diluted 1:1 in Matrigel:phosphate-buffered saline (PBS; total number of tumors expected was 10). At the experimental end point, tumors, if existing, were snap-frozen for further molecular analysis. For orthotopic experiments, at least 5-week-old NOD scid gamma mouse (NSG) mice obtained from Charles River Laboratories were used, and the experiment was carried out as previously described (*23*). Briefly, 2 million cells diluted 1:3 in Matrigel:DMEM were injected in the kidney capsule. Four mice were injected per experimental condition. Tumor initiation and growth were monitored by In Vivo Imaging System (IVIS) bioluminescence imaging (PerkinElmer) in both in vivo experiments. The injection area was selected at time 0 for the xenografts and at 24 hours for the orthotopic experiments, and the average signal of all the mice per condition normalized to this initial time point was used for the analysis.

#### Immunohistochemistry staining

Kidneys were collected, fixed overnight with neutral formalin 4%, and washed with PBS and 70% ethanol for 15 min. Lungs were embedded in paraffin, sectioned, and stained with hematoxylin and eosin by the human research tissue bank and histopathology research support group from the Cambridge University Hospitals–NHS Foundation. Different kidney sections were imaged using a Slidescanner microscope (Hamamatsu S360).

### Patient samples

Patient samples were obtained by M.G.B.T. at the Royal Free Hospital or E.R.M. upon informed consent for genetic studies and with evaluation and approval from the corresponding ethics committees in accordance with the Declaration of Helsinki (NHS REC 16-WS-0039, South Birmingham Research Ethics Committee).

### Genome-wide CRISPR-Cas9 screen and data analysis

Cas9-expressing cells were generated, and the lentiviral gRNA library was produced using HEK293FT cells as described above. A total of 150 million cells were transduced with the pooled library at a low multiplicity of infection (<0.3) to ensure that >85% of cells had a single gRNA integration, resulting in at least 500× gRNA representation. Medium containing the lentiviral particles was removed from the cells the following day, and puromycin selection was applied for the following 3 days. Cells were then propagated for 18 days, and at least 90 million cells were harvested at the end of the assay for genomic DNA extraction. DNA was extracted using the QIAamp DNA Mini Kit (Qiagen) and amplified for the region containing the gRNAs, followed by high-throughput, 19–base pair single-end sequencing (Illumina-C HiSeq 2500).

Guides were quantified against the Yusa Mouse V2 library (Addgene no. 67988) using crisprReadCounts v1.3.1 (https://github.com/cancerit/crisprReadCounts). Raw count normalization to plasmid and copy number correction were performed using pyCRISPRcleanR version 2.0.8 (https://github.com/cancerit/pyCRISPRcleanR). The corrected counts were used as inputs for pairwise comparisons with MAGeCK version 0.5.9.2 to identify significantly enriched and depleted guides or genes using “mageck test” with normalization disabled (`--norm-method none`) (https://sourceforge.net/projects/mageck) (*34*). Quality control and postanalysis tables and plots were generated in RStudio v1.2.1578 (R version 3.6.1). The detailed R scripts for the screen analysis are available on GitHub (https://github.com/team113sanger/Fumarate_Hydratase_FH_CRISPR) and at https://doi.org/10.5281/zenodo.6986346. Raw data can be found in www.ebi.ac.uk/ena/browser/home under the following accession numbers: ERS3957541, ERS3957542, ERS3957543, ERS3957544, ERS3957545, ERS3957546, ERS3957547, ERS3957548, ERS3957549, and ERS3957550.

### Cellular and molecular assays

#### Immunofluorescence

Immunofluorescence was performed as previously described (*35*). Briefly, cells were fixed with 4% paraformaldehyde in PBS for 15 min and then washed three times with PBS. Cells were permeabilized with 0.3% Triton X-100 in 4% bovine serum albumin (BSA) for 20 min, followed by three PBS washes. Cells were then blocked with 4% BSA for 30 min, followed by incubation with primary antibodies in 4% BSA overnight at 4°C. After five washes with PBS, cells were incubated with appropriate secondary antibodies (1:1000) for 2 hours at room temperature. After three washes in PBS, coverslips were mounted onto slides using the ProLong Gold antifade mountant (Thermo Fisher Scientific).

#### Invasive growth, transwell migration, and wound healing assays

The invasive growth assay was performed as previously described (*35*). Briefly, cells (1000 cells per drop) were maintained in drops (25 μl per drop) with DMEM and 20% methylcellulose (Sigma-Aldrich, M0387) on the cover of a 100-mm culture plate. Drops were incubated at 37°C and 5% CO_2_ for 72 hours. Once formed, spheroids were collected, resuspended in collagen I solution (Advanced BioMatrix PureCol), and added to 24-well plates. After 4 hours, DMEM was then added on top of the well, and to calculate the increased spheroid area, pictures were taken using an EVOS microscope (Thermo Fisher Scientific) at days 0 and 2. For invasive growth quantification, an increase in the area between day 0 and day 2 was calculated using FiJi software. The migration transwell assay was performed as previously described (*35*). In brief, chambers with membranes of 8-μm pores (BD Falcon) were used. Cells (50,000 cells per well) were resuspended in 0.1% FBS DMEM and seeded in the upper part of the chamber. In the bottom part of the well, 1.4 ml of complete DMEM was added. Plates were maintained at 37°C and 10% CO_2_ for 24 hours. Migration was stopped washing the wells twice with PBS and using a cotton bud to remove the remaining cells of the upper part of the membrane, being careful not to compromise it. The membrane was fixed with 10% formalin (15 min at 4°C) and stained with crystal violet. Cells were counted under the EVOS microscope (Thermo Fisher Scientific). For wound healing assays, well inserts creating the barrier were purchased from Abcam (ab242285). Cells were seeded and cultured until a monolayer was formed at both sides of the barrier. The insert was removed, and cells were monitored for 24 hours. After that, the cells were washed once with PBS and fixed with 10% buffered formalin. The wound field distance, which directly correlates with the migration capacity of the cells, was imaged using the EVOS microscope (Thermo Fisher Scientific) and measured using FiJi software.

#### *Cell growth, BrdU incorporation,* β*-galactosidase assay, and cell cycle analysis*

Cell proliferation was analyzed using the Incucyte SX5 by means of phase-contrast sharpness for 4 to 6 days or through crystal violet staining as previously described (*36*). Briefly, 5000 cells were plated onto 24-well plates (at least three replicates/experimental conditions for each cell line), and at each time point, cells were washed with PBS and fixed with 4% buffered formalin. Once all the time points were collected, the cells were washed with PBS and incubated with 0.1% crystal violet diluted in 20% methanol. Once the cells were stained, the plates were washed with water and dried overnight. To quantify the staining differences, cells were diluted in 0.5 ml of 10% acetic acid for 30 min at room temperature and quantified using a TECAN spectrophotometer reading the absorbance at 595 nm. The senescence assay was performed using a senescence β-galactosidase staining kit from Cell Signaling Technology (no. 9860) following the manufacturer’s instructions. BrdU staining was performed as previously described (*37*). For BrdU incorporation, cells were seeded on coverslips in 12-well plates, and after 2 days, cells were incubated with BrdU (3 μg/ml; Sigma-Aldrich). Cells were fixed with 4% paraformaldehyde, permeabilized with 1% Triton X-100, and incubated with a monoclonal anti-BrdU antibody (ab6326) at a 1:100 dilution. Images were obtained using an SP5 confocal microscope. At least three different areas per coverslip were quantified. Cell cycle analysis was performed using propidium iodide staining. Two hundred thousand cells per well were seeded in six-well plates and grown for 2 days. Cells were collected, resuspended in 1 ml of PBS, and fixed while vortexing, adding drop by drop 2.5 ml of cold absolute ethanol. Cells were stored at −20°C overnight. The next day, samples were centrifuged and washed once with PBS. Cell pellets were then resuspended in propidium iodide (1 μg/ml; ab14083) solution with 0.05% Triton X-100 and ribonuclease (25 μg/ml; Thermo Fisher Scientific, 12091021). Samples were analyzed using an LSRFortessa (BD Biosciences) FACS.

### RNA extraction and transcriptomic analysis

For RNA assays, 300,000 cells were plated onto a six-well plate. The day after, cells were washed in PBS, and then RNA was extracted using an RNeasy kit (Qiagen) following the manufacturer’s protocol. RNA was eluted in water and then quantified using NanoDrop (Thermo Fisher Scientific). One microgram of RNA was reverse-transcribed using the Quantitect Reverse Transcription Kit (Thermo Fisher Scientific). For real-time quantitative polymerase chain reaction, complementary DNA was run using TaqMan assay primers (Thermo Fisher Scientific) and TaqMan Fast 2X master mix (Thermo Fisher Scientific). β-Actin was used as the endogenous control for in vitro and in vivo experiments and Ribosomal Protein Lateral Stalk Subunit P0 (RPLP0) for patient samples. The different biological replicates and gene expression differences were analyzed using the Δ*C*t formula. For tissue samples, a maximum of 30 mg per tissue were homogenized using the Precellys tissue homogenizer. The RNA extraction was performed with the RNeasy Kit (Qiagen) following the manufacturer’s instructions, and gene expression analysis was performed as previously mentioned. The references of primers used are in table S1. For RNA-seq sample preparation, RNA was extracted as mentioned before and further purified using the RNA Clean and Concentrator Kit (Zymo Research). The RNA-seq was done on a single-end run using the TruSeq Stranded mRNA from Illumina, and the library preparation was performed following the manufacturer’s instructions. The sequencing was done on a 75-cycle high-output NextSeq 500 kit. The differential gene expression analysis was done using the counted reads and the R package edgeR version 3.26.5 (R version 3.6.1) (*38*). Before running EdgeR, genes that were filtered did not have at least 5 cpm in at least half of the samples. Pairwise comparisons were run for Cl19 versus Cl19_gHira, Cl19__VS__Fl, Fl__VS__Fl_gHira using the exact test and adjusted for multiple testing using Benjamini-Hochberg. Raw data from the RNA-seq experiment were deposited in Gene Expression Omnibus (GEO) database under GSE201992 accession number, and processed data can be found in table S2.

### Protein lysates and Western blot

Three hundred thousand cells per well were seeded in six-well plates. The day after, cells were washed in PBS and then lysed on ice with radioimmunoprecipitation assay (RIPA) buffer [80 μl per well; 150 mM NaCl, 1% NP-40, 0.5% sodium deoxycholate, 0.1% SDS, and 25 mM tris] supplemented with protease and phosphatase inhibitors (Protease inhibitor cocktail, Phosphatase inhibitor cocktail 2/3, Sigma-Aldrich) for 5 min. Cells extracts were scraped and further lysed in a roller for 15 min. Protein quantification was done using a bicinchoninic acid assay (BCA) kit (Pierce) following the manufacturer’s instructions. Absorbance was read using the TECAN spectrophotometer at 562 nm. Samples were resuspended in the Bolt Loading buffer 1× (Thermo Fisher Scientific), and 10 to 20 μg of protein were loaded into 4 to 12% Bis-Tris Bolt gel and run at 150 to 200 V constant for 1 hour in Bolt Mops/MES 1× running buffer (Thermo Fisher Scientific). Dry transfer of the proteins to a nitrocellulose membrane was done using IBLOT2 (Thermo Fisher Scientific) for 12 min at 20 V. Membranes were incubated in 5% nonfat milk diluted in tris-buffered saline (TBS) 1× + 0.01% Tween 20 (TBS-T) for 30 min. Primary antibodies were incubated overnight at 4°C. Calnexin antibody was purchased from Abcam (ab22595), Fh1 antibody from Origene (TA500681), anti-DDK (Flag) antibody from Origene (TA50011-30), Myc antibody from Cell Signaling Technologies (18583S), Cdh1 from BD (610181), and Hira WC119 clone from Merck (04-1488).The day after, membranes were washed three times in TBS-T 1× and then secondary antibodies (conjugated with 680- or 800-nm fluorophores; Li-Cor) incubated for 1 hour at room temperature at 1:5000 dilution in 5% nonfat milk diluted in TBS-T. Images were acquired using Image Studio lite 5.2 (Li-Cor) on Odyssey CLx instrument 875 (Li-Cor).

### ChIP sequencing

Cells (2 × 10^7^) were cross-linked in 1% formaldehyde for 10 min at room temperature and quenched with 0.125 M glycine for at least 5 min. Cells were washed twice with 1× PBS and scraped into ice-cold 1× PBS supplemented with protease inhibitor cocktail. Cells were snap-frozen at this point and stored at −80°C until further use. Protein A Dynabeads were blocked and incubated with 2 μg of anti-H3.3 antibody and 1 μg of Spike-in antibody. Cells were lysed in serial rounds of lysis buffer 1, lysis buffer 2, and lysis buffer 3. Chromatin was sonicated for 10 to 15 cycles using a Diagenode Pico and then supplemented with 20 ng of Spike-In Drosophila chromatin. Chromatin was then incubated with beads overnight. Beads were washed five times with RIPA buffer and washed with a final 1× TE wash. DNA was eluted in elution buffer and purified using a DNA clean and concentrator kit. Eluted DNA was quantified using a QuBit. DNA libraries were prepared using a TruSeq ChIP sample prep kit (Illumina) and sequenced on a NextSeq 550 (Illumina) platform. Raw data from the ChIP-seq experiment were deposited in the GEO database under GSE203056 accession number.

For data analysis, sequencing reads were aligned to mm10 and dm6 using Bowtie2 v2.2.3 (*39*). Reads aligning to the Drosophila genome were counted and used to generate scale factors. Binary Alignment Map (BAM) files were then scaled to the sample with the lowest number of *Drosophila* reads. Only reads with a mapping quality >q30 were retained. Replicates were merged, and peak calling was performed using Model-based Analysis of ChIP-seq (MACS2) v2.1.1 (*40*) using default parameters with additional –SPMR parameter. bedGraph files were converted to bigwig using BedGraphtoBigWig script and visualized in the Integrative Genomic Viewer (IGV) Genome Browser.

Metagene tag density plots were generated using computeMatrix and plotProfile tools from the deepTools package (*41*). Correlation of biological replicates was visualized using multiBigwigSummary and plotCorrelation from the deepTools package.

### Metabolomics

Cells were seeded at a confluency of 60% per well in six-well plates. The following day, the metabolomics extraction was performed after quickly washing the cells with PBS twice and adding the extraction buffer [50% Liquid chromatography–mass spectrometry (LC-MS)–grade methanol, 30% LC-MS–grade acetonitrile, 20% ultrapure water, and 5 μM valine-d8]. Cells were collected and kept for 15 min in a shaker at 4°C. After this, samples were centrifuged during 20 min at maximum speed, and the supernatant was collected for further analysis. Hydrophilic interaction liquid chromatography (HILIC) chromatographic separation of metabolites was achieved using a Millipore Sequant ZIC-pHILIC analytical column (5 μm, 2.1 mm by 150 mm) equipped with a 2.1 mm by 20 mm guard column (both 5-mm particle size) with a binary solvent system. Solvent A was 20 mM ammonium carbonate and 0.05% ammonium hydroxide; solvent B was acetonitrile. The column oven and autosampler tray were held at 40° and 4°C, respectively. The chromatographic gradient was run at a flow rate of 0.200 ml/min as follows: 0 to 2 min, 80% B; 2 to 17 min, linear gradient from 80% B to 20% B; 17 to 17.1 min, linear gradient from 20% B to 80% B; 17.1 to 22.5 min, hold at 80% B. Samples were randomized and analyzed with LC-MS in a blinded manner with an injection volume of 5 μl. Pooled samples were generated from an equal mixture of all individual samples and analyzed and interspersed at regular intervals within sample sequence as a quality control.

Metabolites were measured with a Thermo Fisher Scientific Q Exactive Hybrid Quadrupole-Orbitrap Mass spectrometer (HRMS) coupled to a Dionex Ultimate 3000 ultrahigh-performance liquid chromatography. The mass spectrometer was operated in full-scan, polarity-switching mode, with the spray voltage set to +4.5 kV/−3.5 kV, the heated capillary held at 320°C, and the auxiliary gas heater held at 280°C. The sheath gas flow was set to 55 units, the auxiliary gas flow was set to 15 units, and the sweep gas flow was set to 0 unit. HRMS data acquisition was performed in a range of mass-to-charge ratio (*m/z*) = 70 to 900, with the resolution set at 70,000, the Automatic Gain Control (AGC) target at 1 × 10^6^, and the maximum injection time at 120 ms. Metabolite identities were confirmed using two parameters: (i) Precursor ion *m/z* was matched within 5 parts per million (ppm) of theoretical mass predicted by the chemical formula, and (ii) the retention time of metabolites was within 5% of the retention time of a purified standard run with the same chromatographic method. Chromatogram review and peak area integration were performed using the Thermo Fisher Scientific software Tracefinder 5.0, and the peak area for each detected metabolite was normalized against the total ion count of that sample to correct any variations introduced from sample handling through instrument analysis. The normalized areas were used as variables for further statistical data analysis. Raw data and normalized data to total ion count can be found in table S4 and in the corresponding accession DOI: http://dx.doi.org/10.21228/M8QM6W.

### Statistical analysis and coding

*n* values represent the number of independent experiments performed or the number of individual mice. For each independent in vitro experiment, at least three biological replicates were used (except from Western blots where technical replicates are represented), and a minimum number of three experiments were done to ensure adequate statistical power. For in vitro experiments, Student’s *T* test was applied for two component comparisons. For in vivo experiments, nonparametric Mann-Whitney exact test was used. Statistical analyses involving fold changes were analyzed using the one-sample *T* test with a null hypothesis of 1. Two-tailed *t* test statistical analysis was used when testing for differences between two conditions. Error bars displayed on graphs represent the ± SEM. All statistical analyses were performed using GraphPad PRISM 9 software. Figures were compiled using either Adobe Illustrator or BioRender. The KIRP patient survival analysis was performed using GEPIA with the overall survival and median cutoff of 50% (http://gepia.cancer-pku.cn) (*26*). KIRP II patient gene expression counts were downloaded from The Cancer Genome Atlas (TCGA) based on patient identifiers from Chen *et al.* (*42*). Samples were stratified on the basis of FH expression into two groups {low = bottom 25% [log_2_(counts + 1) <= 11.8], high => 25% [log_2_(counts + 1) > 11.8]}. Code and data are available at https://github.com/ArianeMora/KIRP_PE2 and under DOI: https://doi.org/10.5281/zenodo.6984853.

GSEA was performed by recapitulating on the gene sets published on the Molecular Signatures Database using the packages fgsea (v1.20.0) and GSEABase (v1.56.0) (*43*–*45*) (https://bioconductor.org/packages/release/bioc/html/GSEABase.html). The EMT gene set was generated by manually curating the gene list published by Taube *et al.* (*46*). All gene names were translated into mouse gene names before the analysis using scibiomart (v. 1.0.2), a wrapper around the API from BioMart. Plots were generated using the EnhancedVolcano package (v.1.12.0) (https://github.com/kevinblighe/EnhancedVolcano). Detailed code can be found under https://github.com/ChristinaSchmidt1/Oncogenic_events_in_HLRCC. RNA-seq data from HLRCC patients’ primary tumors and paired adjacent tissue were downloaded from the GEO database (GSE157256) analyzed as described under https://github.com/ChristinaSchmidt1/Oncogenic_events_in_HLRCC. In brief, differential expression analysis was performed using DESeq2 (v1.34.0) (*47*) and GSEA was performed as previously described (*43*–*45*). The EMT gene set was generated as described before (*46*). Plots were generated using the EnhancedVolcano package (v1.12.0) (https://github.com/kevinblighe/EnhancedVolcano).

For the TF analysis, we used the normalized count data from EdgeR (for details, see the RNA-seq analysis) as the input. Moreover, we use Dorothea (v.1.6.0), a mouse TF regulon collection, filtering for confidence = “A” (*48*, *49*) (https://bioconductor.org/packages/release/data/experiment/html/dorothea.html). DoRothEA is a gene regulatory network containing signed TF, target gene interactions. DoRothEA regulons, the collection of a TF and its transcriptional targets, were curated and collected from different types of evidence for both human and mouse. A confidence level was assigned to each TF-target interaction based on the number of supporting evidence. The TF analysis is performed using dorothea’s function “run_viper,” which is based on the viper package (v.1.28.0) (*50*). To compare the conditions of interest, we calculated the mean of the biological replicates and calculated the TF change by subtracting one condition from the other. The *P* value was calculated using the *t* test. Detailed data and code information can be found at https://github.com/ChristinaSchmidt1/Oncogenic_events_in_HLRCC, under https://doi.org/10.5281/zenodo.6984751 and in table S5.

## Notes

View/request a protocol for this paper from *Bio-protocol*.
