# Supplementary material for: HIRA loss transforms FH-deficient cells
Source: Sci Adv. 2022 Oct 21;8(42):eabq8297. doi: 10.1126/sciadv.abq8297 (PMC9586478; doi:10.1126/sciadv.abq8297)

Science Advances
AAAS

Supplementary Materials for

HIRA loss transforms *FH*-deficient cells

Lorea Valcarcel-Jimenez *et al.*

Corresponding author: Christian Frezza, christian.frezza@uni-koeln.de

Sci. Adv. **8**, eabq8297 (2022)
DOI: 10.1126/sciadv.abq8297

The PDF file includes:

Figs. S1 to S8
Legends for tables S1 to S5

Other Supplementary Material for this manuscript includes the following:

Tables S1 to S5

Supplementary Figure 1. *Fh1* loss in mouse epithelial kidney cells does not activate a canonical senescence phenotype.

(**a**) Immunoblot of Fh1 expression in *Fh1*-proficient (*$Fh1^{fl/fl}$*), -deficient (*$Fh1^{-/-CL1}$* and *$Fh1^{-/-CL19}$*) and -reconstituted (*$Fh1^{-/-CL1}$ +pFH)* cell lines (One representative blot shown out of >5). **(b)** Representation of β-Galactosidase activity assay images for all cell lines (one representative experiment shown out of 3). No blue (β-Galactosidase activity-positive) cells were observed in comparison with a positive senescent cell line (A375 cells treated with Cisplatin). **(c)** qRT-PCR showing expression levels for *Fh1*, *Cdkn1a* (p21) and *Cdkn2a* (p16) (n=3). β-Actin was used as a housekeeping gene. **(d)** 2D growth of control cells (*$Fh1^{fl/fl}$*) with/without monomethylfumarate (MMF) treatment (400µM). Values normalized to day 0 (n=3). Statistics performed comparing the values of the last time point. **(e)** Cell cycle analysis by means of percentage of PI staining in the S phase of the cell cycle of cells treated with MMF (n=3). **(f)** Representative images of β-Galactosidase activity in cells treated with MMF (one experiment shown out of 3). No blue (β-Galactosidase activity-positive) cells were observed. **(g)** Representative images of spheroids included in collagen I matrix at 0 and 48 hours from *Fh1* -proficient (*$Fh1^{fl/fl}$*), -deficient (*$Fh1^{-/-CL1}$* and *$Fh1^{-/-CL19}$*) and -reconstituted (*$Fh1^{-/-CL1}$ +pFH)* cell lines. One representative experiment shown out of 3. Yellow lines highlight the spheroid area. Error bars represent standard error of the mean (S.E.M). Statistic tests performed: two-tailed Student T test (c), one-tail Student t-test (d, e). Numbers represent p-value for all comparisons.

Supplementary Figure 2. Validation of *Hira* loss in *Fh1*-proficient and -deficient cells.

(a) qRT-PCR showing expression levels for *Hira* and *Fh1* (n=3) in *Fh1*-proficient (*$Fh1^{fl/fl}$ Cas9),* -deficient (*$Fh1^{-/-CL1}$Cas9* and *$Fh1^{-/-CL19}$Cas9*) and -reconstituted (*$Fh1^{-/-CL1}$ +pFH Cas9)* cells alone or under *Hira* depletion (*g1Hira*) (n=3) together with immunoblot for Hira and Fh1 expression in *Fh1*-proficient (*$Fh1^{fl/fl}$ Cas9),* -deficient (*$Fh1^{-/-CL1}$ Cas9* and *$Fh1^{-/-CL19}$ Cas9*) and - -reconstituted (*$Fh1^{-/-CL1}$ +pFH Cas9)* cells alone or under *Hira* depletion (*g1Hira*) (One representative blot out of 3) **(b)** qRT-PCR showing expression levels for *Nqo1* and fumarate abundance in *Fh1*-proficient (*$Fh1^{fl/fl}$ Cas9),* -deficient (*$Fh1^{-/-CL1}$ Cas9* and *$Fh1^{-/-CL19}$ Cas9*) and -reconstituted (*$Fh1^{-/-CL1}$ +pFH Cas9)* cells alone or under *Hira* depletion (*g1Hira*) (n=3 in both cases). (**c**) 2D growth analysis of *Fh1*-proficient (*$Fh1^{fl/fl}$*) and -reconstituted (*$Fh1^{-/-CL1}$ +pFH)* cell lines alone or under *Hira* loss (*g1Hira*) (n=3). Data normalised to time 0. Statistics performed comparing the values of the last time point. **(d)** Cell cycle analysis by means of percentage of cells in the S phase of cell cycle of *Fh1*-deficient cells (*$Fh1^{-/-CL1}$ Cas9* and *$Fh1^{-/-CL19}$ Cas9*) alone or under *Hira* deficiency (*g1Hira*) (n=4). **(e)** DNA synthesis analysis by means of % of BrdU incorporation in *Fh1*-deficient cells (*$Fh1^{-/-CL1}$ Cas9* and *$Fh1^{-/-CL19}$ Cas9*) and *Hira* and *Fh1*-

deficient cells (*Fh1*$^{-/-CL1}$ *g1Hira* and *Fh1*$^{-/-CL19}$*g1Hira*) (n=3). (**f**) Trans-well migration analysis for 24 hours in *Fh1*-proficient and *Fh1*-deficient cells alone or upon *Hira* loss (*g1Hira*) (n=3). Data normalized to initial number of cells. (**g**) Wound healing assay in *Fh1*-proficient and *Fh1*-deficient cells alone or upon *Hira* loss (*g1Hira*) (n=3). The wound distance was measured at 24 hours and normalised to time 0 (**h**) Representative images of spheroids included in collagen I matrix at 0 and 48 hours from *Fh1*-deficient cells (*Fh1*$^{-/-CL1}$ *Cas9, Fh1*$^{-/-CL19}$ *Cas9)* and *Hira* and *Fh1*-deficient cells (*Fh1*$^{-/-CL1}$ *g1Hira, Fh1*$^{-/-CL19}$ *g1Hira)* (n=3). Error bars represent standard error of the mean (S.E.M). Statistic tests performed: one-tailed Student T test. Numbers represent p-value for all comparisons.

Supplementary Figure 3. Validation of *Hira* loss in *Fh1*-proficient and -deficient cells using a second gRNA (*g4Hira*) and effect of Hira reconstitution.

(**a**) qRT-PCR showing expression levels for *Hira* in *Fh1*-deficient cells (*Fh1*$^{-/-CL1/CL19}$ *Cas9)* with a second gRNA (*g4Hira*) together with an immunoblot showing Hira protein levels (One representative blot out of 3). **(b)** Effect of Hira loss with a second gRNA (*g4Hira*) in two *Fh1*-deficient cells (*Fh1*$^{-/-CL1/CL19}$ *Cas9)* on the cell cycle progression by means of percentage of cells in the S phase (n=4). (**c**) 2D growth analysis of two *Fh1*-deficient cells (*Fh1*$^{-/-CL1/CL19}$ *Cas9)* and two *Hira* and *Fh1*-deficient cells (*Fh1*$^{-/-CL1/CL19}$ *g4Hira).* Values normalized to day 0 (n=3). Statistics performed comparing the values of the last time point. **(d)** Wound healing assay by means of wound distance during 24 hours in two *Hira* and *Fh1*-deficient cell lines (*Fh1*$^{-/-CL1/CL19}$ *g4Hira*) (n=3). Data normalized to time 0. (**e**) Immunoblots for Hira reconstitution in four different *Hira* and *Fh1*-deficient cell lines (*Fh1*$^{-/-CL1/CL19}$ *g1Hira and Fh1*$^{-/-CL1/CL19}$ *g4Hira)* and the corresponding signal quantification normalized by the housekeeping gene (Calnexin) (One representative blot out of 3). (**f**) 2D growth at day 4 of four different *Hira* and *Fh1*-deficient cell lines (*Fh1*$^{-/-CL1/CL19}$ *g1Hira and Fh1*$^{-/-CL1/CL19}$ *g4Hira)* transduced with the control plasmid (pCtrl) or with the Hira cDNA plasmid (pHira) (n=3). Error bars represent standard error of the mean (S.E.M). Statistic tests performed: one-tailed Student T test. Numbers represent p-value for all comparisons.

Supplementary Figure 4. Effect of *Hira* and *Fh1* loss *in vivo*.

(**a**) qRT-PCR showing expression levels for *Fh1* and *Hira* in tumour xenografts from control (*Fh1*$^{fl/fl}$ *Cas9)*, *Fh1*-deficient (*Fh1*$^{-/-CL19}$*Cas9)* and *Hira* and *Fh1*-deficient cells (*Fh1*$^{-/-CL1}$ *g1Hira* and *Fh1*$^{-/-CL19}$ *g1Hira)* (n=10 for all conditions except for *Fh1*$^{fl/fl}$ *Cas9*, n=4). No tumours were extracted from the other *Fh1*-deficient cell line (*Fh1*$^{-/-CL1}$*Cas9).* β-Actin was used as a housekeeping gene. **(b)** Images from kidneys injected with *Fh1*-deficient (*Fh1*$^{-/-CL1}$ *Cas9)* and *Hira* and *Fh1*-deficient cells (*Fh1*$^{-/-CL1}$ *g1Hira)* and BioLuminescence Imaging (BLI) and flux

intensity at the experimental end point (week 9). **(c)** Representative H&A staining image of kidney capsule injected with *Fh1*-deficient cells (*Fh1$^{-/-CL1}$ Cas9).* Scale bar represents 1mm. **(d)** Representation of luminescence signal by means of BioLuminiscence Imaging (BLI) and flux intensity normalised to time 0 of cells (*Fh1$^{fl/fl}$ Cas9* and *Fh1$^{-/-CL1}$ Cas9)* injected in the flanks of the mice (n= 10 tumours). **(e)** Representation of luminescence signal by means of BioLuminiscence Imaging (BLI) and flux intensity normalised to day 1 (day after surgery) of *Fh1$^{fl/fl}$ Cas9* and *Fh1$^{-/-CL1}$ Cas9* cells injected in the kidney capsule (n=4 kidneys/condition). **(f)** qRT-PCR showing expression levels for *FH* and *HIRA* for two HLRCC patient samples and three adjacent normal tissue samples (n= 3 Normal, n=2 HLRCC). RPLP0 was used as a housekeeping gene. Error bars represent standard error of the mean (S.E.M). Statistic tests performed: two-tailed Mann-Whitney U test (a, d, e). Numbers represent p-value for all comparisons. No statistics were performed in panel f due to n<3.

Supplementary Figure 5. Transcriptomic changes mediated by *Hira* and *Fh1* loss-EMT signature.

(**a**) Volcano plot representing the GSEA data from *Fh1$^{-/-CL19}$ Cas9* vs *Fh1$^{fl/fl}$ Cas9.* The specific databases are colour coded as indicated**. (b)** Volcano plot representing the GSEA from *Fh1$^{fl/fl}$ g1Hira vs Fh1$^{fl/fl}$ Cas9*. The specific databases are colour coded as indicated. **(c)** Volcano plots of the genes present in HALLMARK MYC_Targets_V1 and E2F_Targets signatures for *Fh1$^{fl/fl}$ g1Hira vs Fh1$^{fl/fl}$ Cas9* cells. **(d)** Immunoblots of Hira and Cdh1 in *Fh1*-deficient cells and *Hira* and *Fh1*-deficient cells (*g1 and g4Hira*). Calnexin and β-Actin were used as housekeeping genes. One representative experiment shown out of 3. **(e)** qRT-PCR showing expression levels for *Cdh1* and *Vim* in *Fh1*- proficient and -deficient cells and in *Hira* and *Fh1*-deficient cells (*g1Hira*) (n=4). **(f)** qRT-PCR showing expression levels for *Cdh1* and *Vim* in *Fh1*-proficient and deficient cells and in *Hira* and *Fh1*-deficient cells (*g4Hira*) (n=3). **(g)** qRT-PCR showing expression levels for *Vim* in xenograft tumours generated in vivo for the corresponding conditions shown (n=10 except *Fh1$^{fl/fl}$ Cas9* n=4). β-Actin was used as a housekeeping gene for the qRT-PCRs. For volcano plots, signatures are highlighted depending on database represented. Error bars represent standard error of the mean (S.E.M). NES=Normalized enrichment score. FC= Fold change. HR= Hazard Ratio. Statistic tests performed: two-tailed Student T test (e,f) and two-tailed Mann-Whitney U test (g). Numbers represent p-value for all comparisons.

Supplementary Figure 6. Transcriptomic changes mediated by *Hira* and *Fh1* loss-MYC/E2F signatures.

(a) Volcano plots of the genes present in the significantly upregulated signatures in the GSEA for HLRCC vs Normal (HALLMARK MYC_Targets_V1 and E2F_Targets). **(b)** qRT-PCR showing expression levels for *Hira, Myc, Kpna2* and *Ppm1d* (MYC targets) in *Fh1*-proficient (*Fh1$^{fl/fl}$ Cas9*) and *Hira*-deficient *Fh1*-proficient cell lines (*Fh1$^{fl/fl}$ g1Hira*) (n=3). β-Actin was used as a housekeeping gene. (**c**) qRT-PCR showing expression levels for *Myc, Kpna2* and *Ppm1d* of *Fh1*-deficient (*Fh1$^{-/-CL1}$*) and *Hira* and *Fh1*-deficient cell lines (*Fh1$^{-/-CL1}$ g4Hira*). Dotted line represents control (*Fh1$^{fl/fl}$ Cas9*) (n=3). **(d)** qRT-PCR showing expression levels for *Myc, Kpna2* and *Ppm1d* in xenograft tumours generated with *Fh1*-proficient, *Fh1*-deficient, and *Hira* and *Fh1*- deficient cells (n=10 for all conditions except for *Fh1$^{fl/fl}$ Cas9* (n=4)**. (e)** Percentage overall survival data associated to E2F targets and MYC targets signatures expression from Papillary type renal cancer (KIRP) using GEPIA(*25*). **(f)** Lollipop chart with the transcription factor analysis from the RNA-Seq data comparing *Fh1*-deficient cells versus *Fh1*-proficient cells. Error bars represent standard error of the mean (S.E.M). Statistic tests performed: two-tailed Student T test (b), one-tailed Student T test and one sample t test with a null hypothesis of 1 (c), two-tailed Mann-Whitney U test for unpaired comparisons and Wilcoxon test for paired comparisons (d). β-Actin used as a housekeeping gene. Numbers represent p-value for all comparisons.

Supplementary Figure 7. ChIP-Seq analysis of *Hira* and *Fh1*-deficient cells.

(a) Correlation of normalized H3.3 ChIP signal with upregulated and downregulated genes in the RNA-Seq results for the comparisons shown. **(b)** Normalized H3.3 ChIP signal associated with EMT signature expression for the comparisons shown. **(c)** IGV snapshot from H3.3 ChIP-Seq for the promoter regions of *Kpna2* and *Ppm1d* genes. TSS=Transcription starting site, TES= Transcription end sites. Shadows represent the S.E.M.

Supplementary Figure 8. Results scheme.

Schematic of the results obtained in this work. *Fh1* loss leads to a cell cycle arrest associated with increased migration and invasion. In this context, *Hira* blocks the accessibility of Myc into the nucleus, inhibiting the activation of its transcriptional programme, through a H3.3-independent mechanism. *Hira* loss in *Fh1*-deficient cells increases proliferation and enhances the invasive properties of the cells *in vitro* and *in vivo*. *Hira* loss in this context allows the binding of Myc in the chromatin and the activation of an oncogenic transcriptional programme allowing full blown transformation.

Supplementary Tables information:

S1. Taqman probes and primers used for qRT-PCR.

S2. RNA-Seq and transcriptomic data generated in the study.

S3. RNA-Seq and transcriptomic data publicly available for HLRCC patients.

S4. Metabolomics data generated in this study.

S5. Transcription factor analysis from RNA-Seq generated in this study.

Supplementary Figure 1

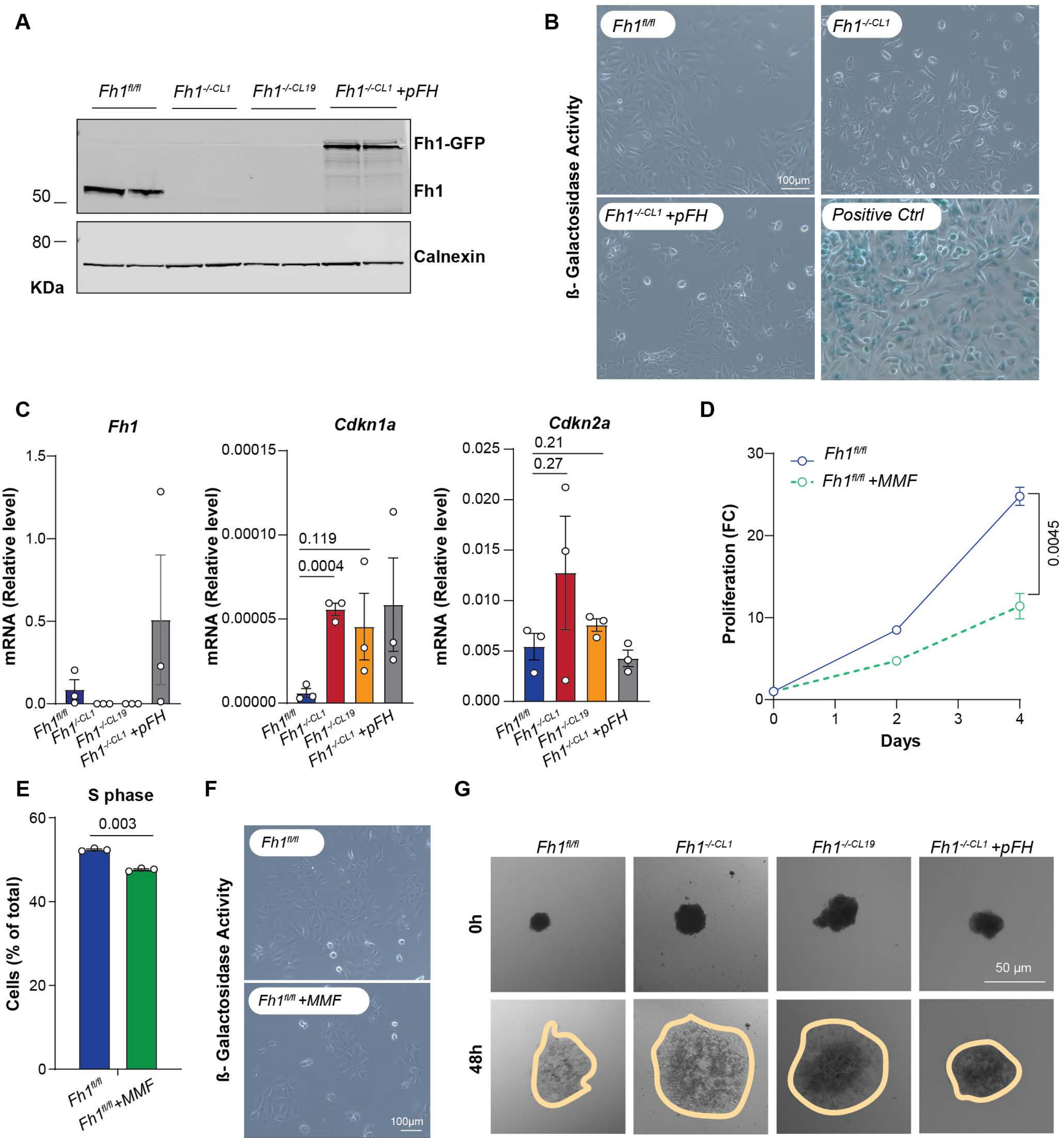

Supplementary Figure 2

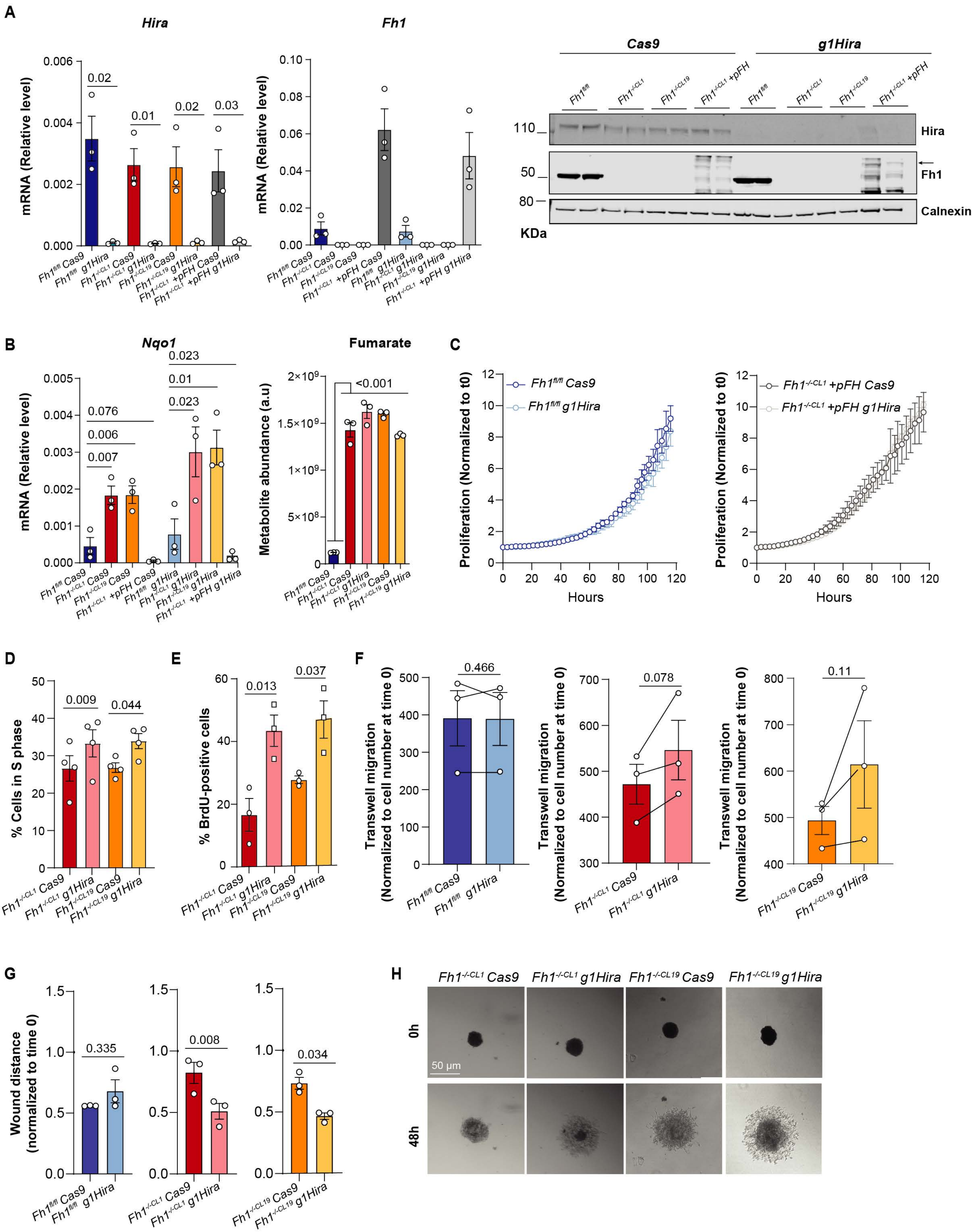

Supplementary Figure 3

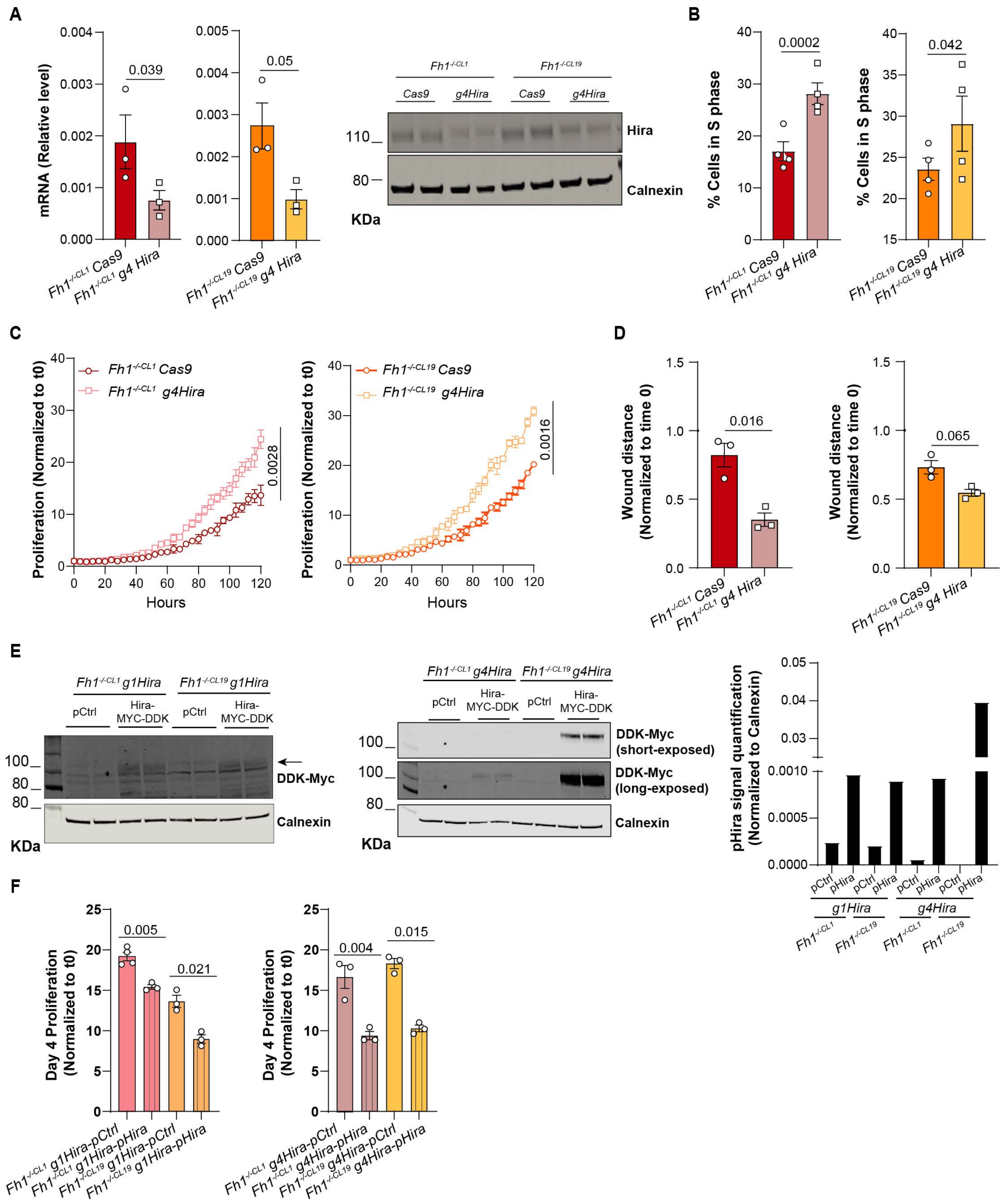

Supplementary Figure 4

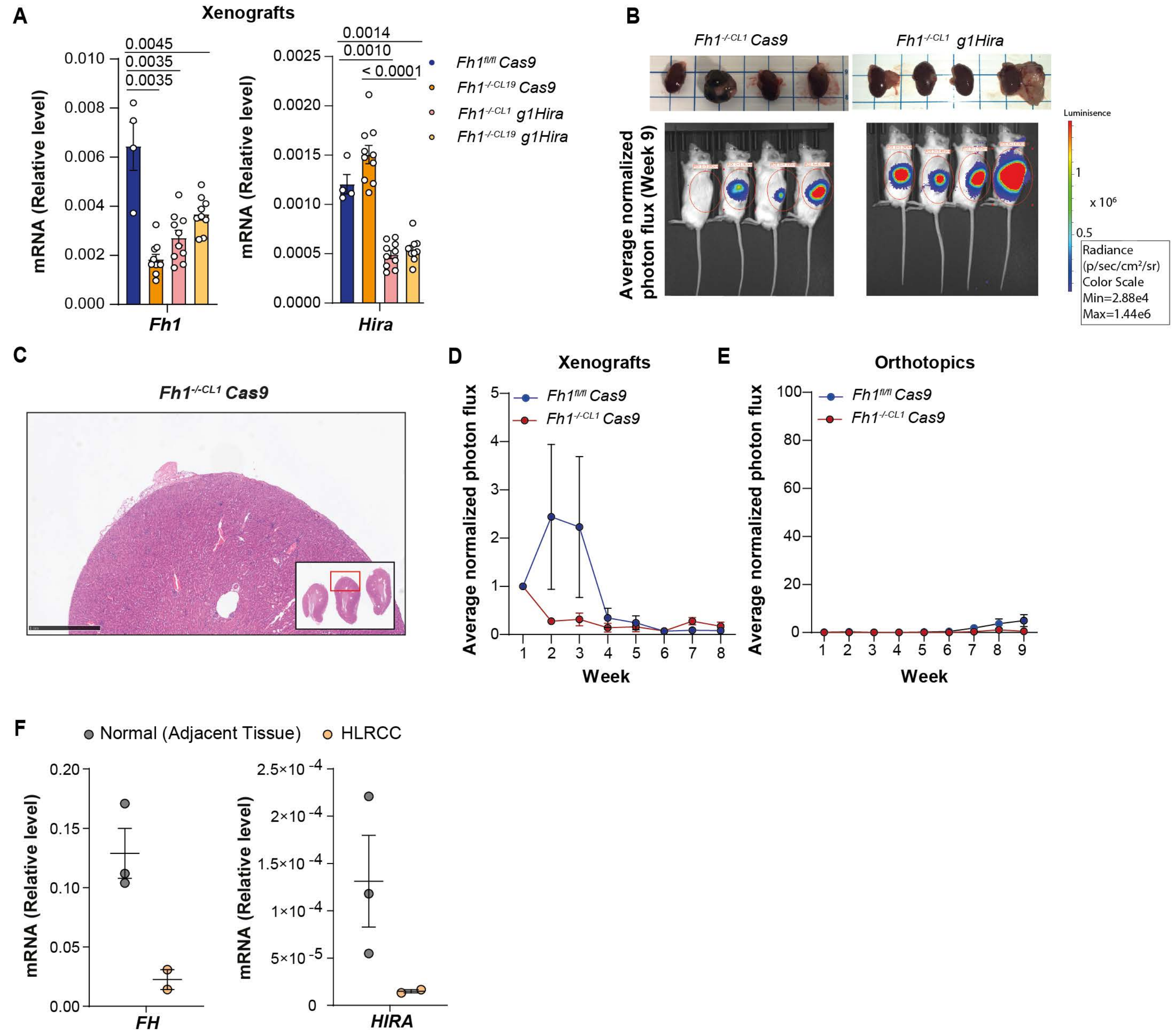

Supplementary Figure 5

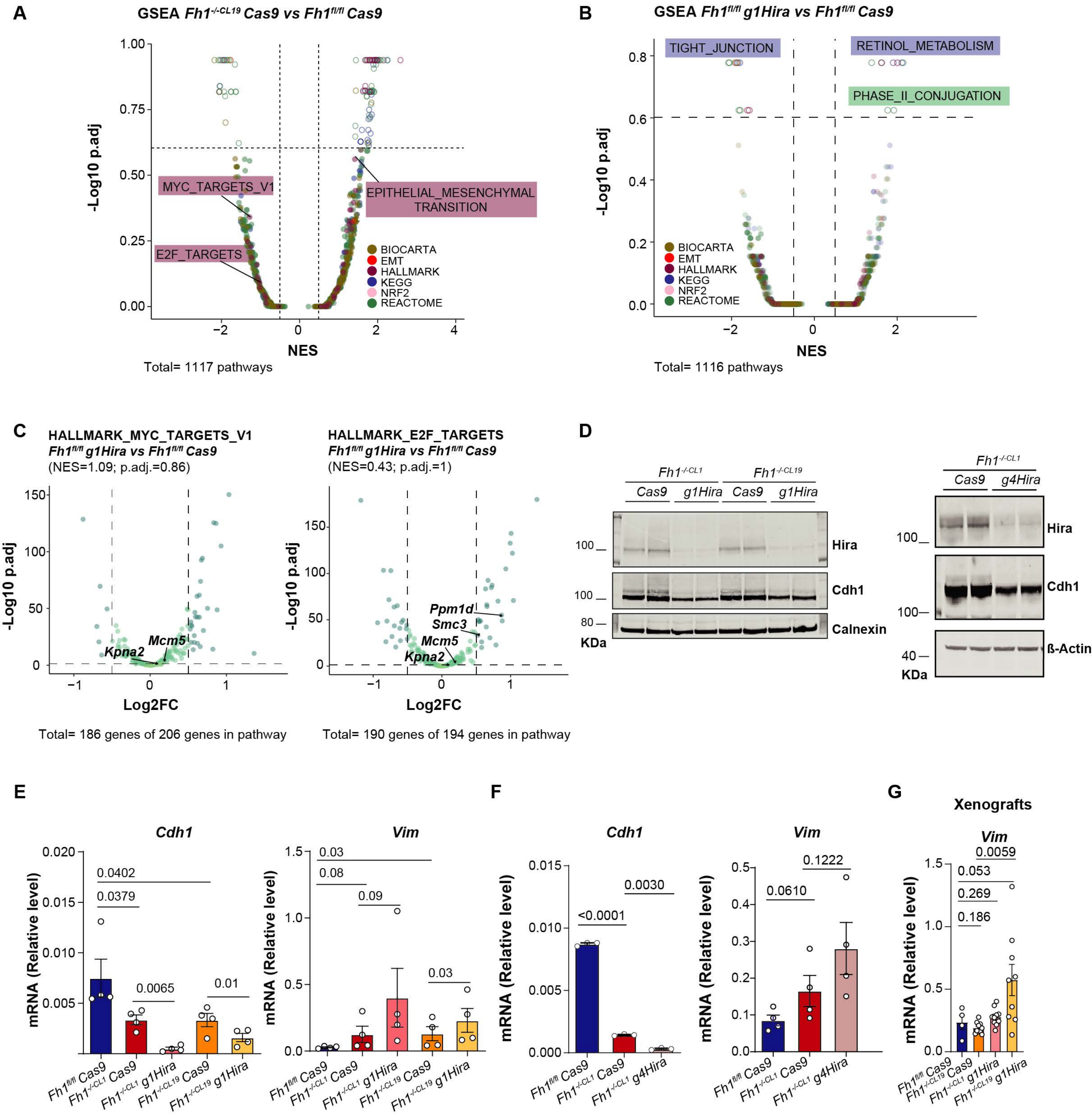

Supplementary Figure 6

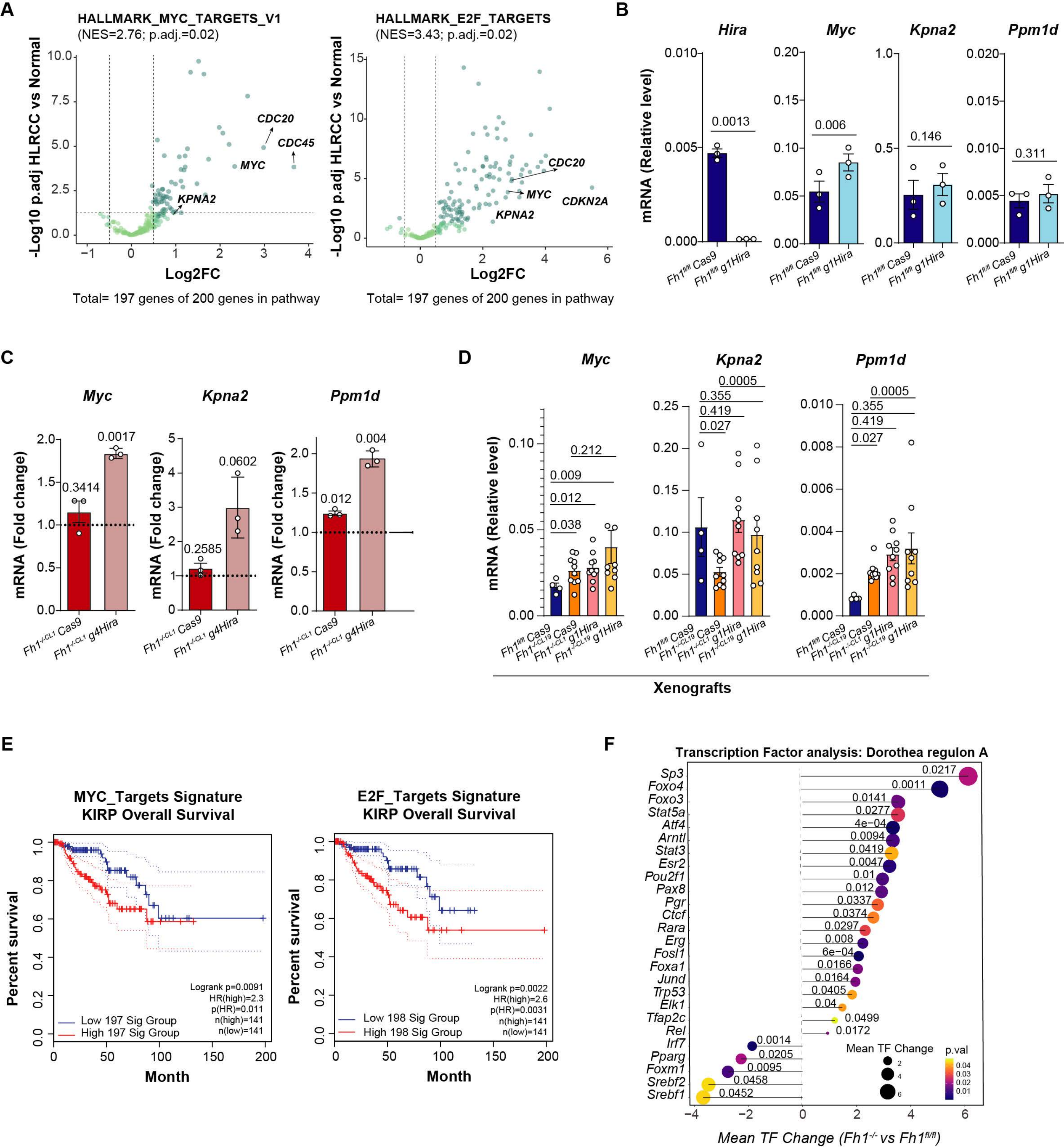

Supplementary Figure 7

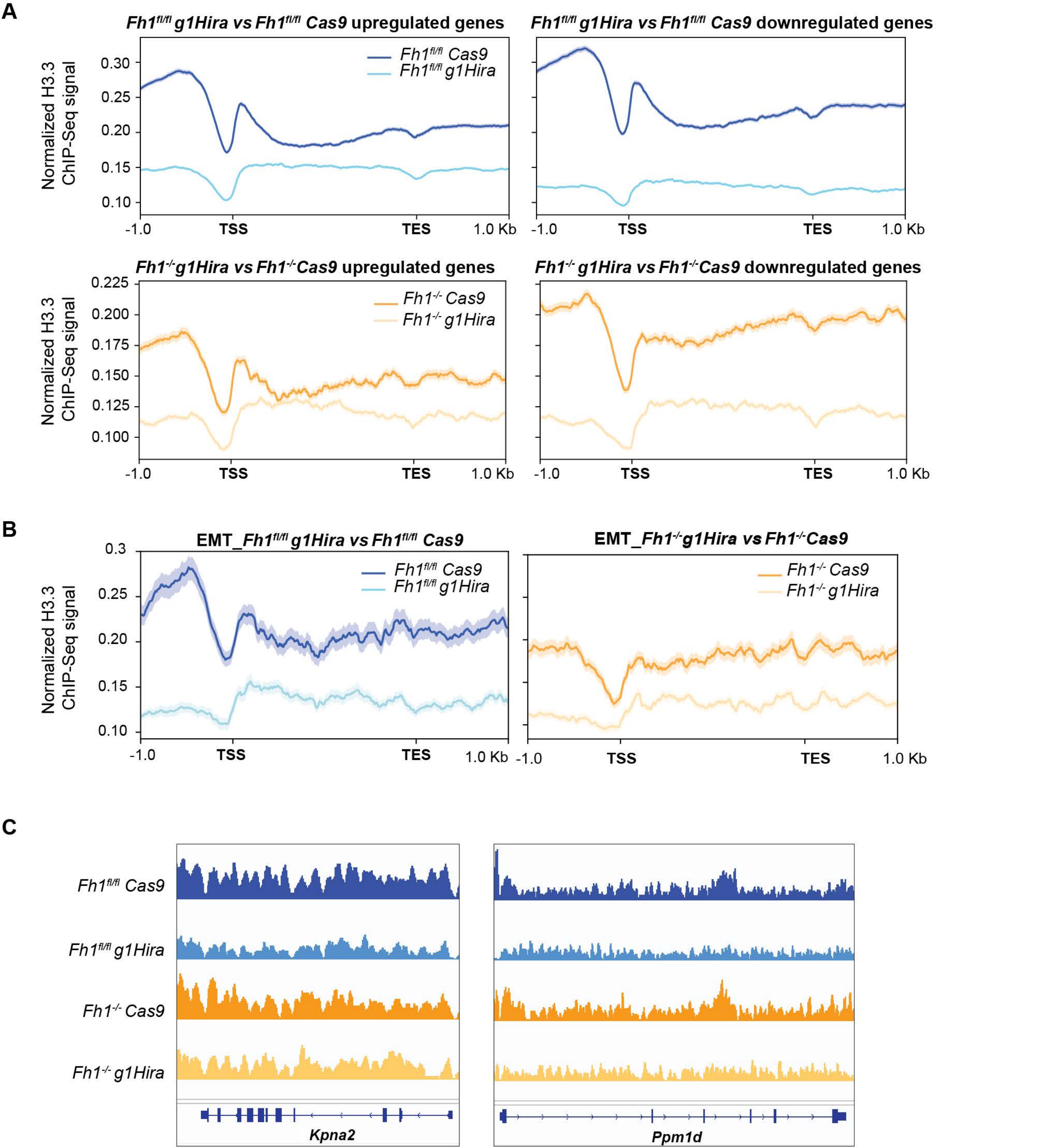

Supplementary Figure 8

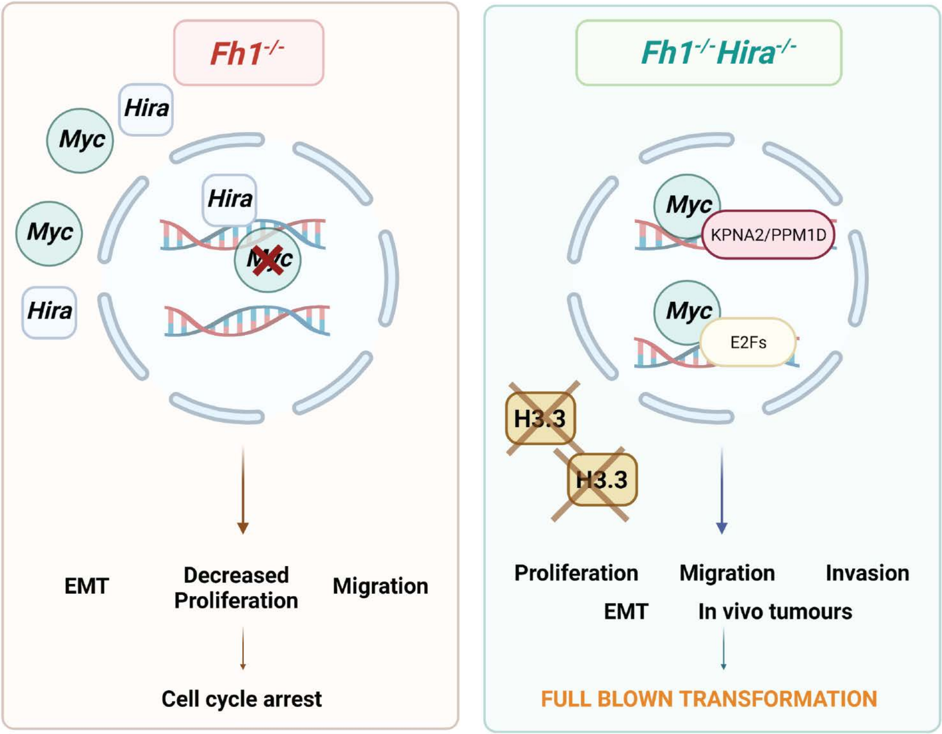

Supplement: Supplementary file 1 — Figs. S1 to S8 [file sciadv.abq8297_sm.pdf]
